# Self-harm with suicidal and non-suicidal intent in young people in sub-Saharan Africa: a systematic review

**DOI:** 10.1186/s12888-020-02587-z

**Published:** 2020-05-14

**Authors:** Emmanuel N-B Quarshie, Mitch G. Waterman, Allan O. House

**Affiliations:** 1 Department of Psychology, University of Ghana, P.O. Box LG 84, Legon, Accra Ghana; 2 School of Psychology, University of Leeds, Leeds, UK; 3 Leeds Institute of Health Sciences, University of Leeds, Leeds, UK

**Keywords:** Adolescents, Attempted suicide, Self-harm, Sub-Saharan Africa, Suicide

## Abstract

**Background:**

Self-harm, whether attributed to suicidal or non-suicidal motives, is associated with several poor outcomes in young people, including eventual suicide. Much of our understanding of self-harm in young people is based on literature from Europe (particularly, the UK), North America, and Australia. We aimed to synthesise the available evidence on prevalence, the commonly reported self-harm methods, correlates, risk and protective factors, and reasons for self-harm, in adolescents (aged 10–25 years) in sub-Saharan Africa.

**Method:**

We searched MEDLINE, PsycINFO, PubMed, African Journals OnLine, and African Index Medicus for records from 1950 through August 2019, without language restrictions. We supplemented the database searches by searching relevant portals for postgraduate theses, reference harvesting, contacting authors for unpublished studies, and hand searching relevant print sources. We applied narrative synthesis to the evidence.

**Results:**

Seventy-four studies from 18 sub-Saharan African countries met the inclusion criteria. The median lifetime prevalence estimate was 10·3% (interquartile range [IQR] 4·6% – 16·1%); median 12-month prevalence estimate was 16·9% (IQR: 11·5% – 25·5%); median 6-month prevalence estimate was 18·2% (IQR: 12·7% – 21·8%); and the median 1-month prevalence estimate was 3·2% (IQR: 2·5–14·8%). Studies from Western sub-Saharan Africa reported the highest 12-month prevalence estimates (median = 24·3%; IQR = 16·9% – 27·9%). Clinical samples commonly reported overdose, whereas self-cutting was most commonly reported in non-clinical samples. Academic failure, sexual, emotional, and physical abuse, romantic relationship problems, family conflict, depression, and previous self-harm were identified as key correlates of self-harm. No study reported protective factors against self-harm.

**Conclusion:**

Variation in estimates was explained by small sample sizes and variation in definitions and measures used. Exploration of associations, risks and protective factors was based upon concepts and measures derived from high income countries. More detailed and culturally sensitive research is needed to understand the context-specific risks and protective factors for self-harm in adolescents in sub-Saharan Africa.

## Background

The World Health Organisation (WHO) defines self-harm as “an act with non-fatal outcome in which an individual deliberately initiates a non-habitual behaviour, that without intervention from others will cause self-harm, or deliberately ingests a substance in excess of the prescribed or generally recognised therapeutic dosage, and which is aimed at realising changes that the person desires via the actual or expected physical consequences” [1, 2].

This definition does not distinguish acts of self-harm according to intent, and for brevity in this review we use the term “self-harm” to refer to acts that are attributed to suicidal and non-suicidal motivations. Self-harm among young people is a recognised problem in the mental health of populations in high income countries, where it is associated with a number of poor outcomes including eventual suicide [3, 4]. By comparison we know little about self-harm in young people in sub-Saharan Africa; instead much of our understanding is based on extrapolation from literature from Europe, particularly the UK, North America, and Australia [5–8]. Earlier regional reviews have either included only a few selected studies of young people from Africa, [9–11] or included only studies involving adult population samples from the region [12]. Thus, we have found no existing review that has systematically appraised the available published and unpublished evidence specifically on self-harm among adolescents in countries in sub-Saharan Africa.

The aims of the present review were to:

1. Describe the lifetime, 12-month, 6-month, and 1-month prevalence estimates of self-harm in young people (aged 10–25 years) across sub-Saharan Africa.
2. Describe the commonest methods of self-harm in young people identified across the previous studies.
3. Identify the commonest associations, risks, and protective factors associated with self-harm in young people observed in previous studies across sub-Saharan Africa.
4. Describe the self-reported reasons for self-harm in young people across sub-Saharan Africa.

## Methods

This systematic review followed PRISMA guidelines [13] (see Additional files 1 and 2). We searched MEDLINE, PsycINFO, PubMed, African Journals OnLine, African Index Medicus, and the South African national Electronic Theses and Dissertations (SA-ETD) portal, (Additional file 3) between January 1950 and August 2019, without language restrictions. The geographic search filter included names of the countries in English and languages relevant to the countries [14]. When a country’s name had changed after 1950 [15], both current and earlier names were included. We searched grey literature, institutional and organisational reports and national and international government reports (e.g., WHO, World Bank, UNICEF, UNDP) [16]. We hand searched edited books, the West African Journal of Medicine, Ghana Medical Journal, South African Medical Journal, Ethiopian Medical Journal, and the East African Medical Journal. We contacted academics with a research interest in self-harm and authors who published two or more articles on self-harm identified during our review (see Additional file 4). Reference harvesting used Science Citation Index, Google Scholar, and manual search. Criteria for inclusion and exclusion of studies are shown in Table 1.

**Table 1:** Summary of inclusion and exclusion criteria

Criterion	Include	Exclude
Definition and measurement of self-harm	▪ Studies with clear definitions of self-harm (or alternative term or concept used) as an intentional act of self-inflicted injury or poisoning, in addition to clear means of case identification, assessment or measurement.	▪ Studies focused on unintended self-harm behaviours (e.g., smoking, drink-driving, eating disorders etc.). ▪ Studies focused on intended self-harm with socially sanctioned motives (e.g., scarification, manhood rituals, ‘body enhancement’, religious fasting, hunger strikes etc.). ▪ Studies focused on intended self-harm behaviours not approved by the broader sociocultural context but are sanctioned by the subcultures (e.g., cult groups, Goth subcultures, Emo subcultures etc.) within which they occur. ▪ Studies focused on suicidal ideations, self-harm thoughts, or threats, as these do not necessarily translate into or represent acts of self-harm. ▪ Studies focused on suicide (self-inflicted death).
Prevalence estimate	▪ Studies with specified time frames within which prevalence of self-harm was assessed.	▪ If prevalence estimates cannot be determined within a clear time frame; ▪ If there is no clear indication of sample size and population denominator.
Setting	▪ Studies with primary focus on self-harm conducted within non-clinical contexts (i.e., general population, community, school-based, households / neighbourhoods, street-connected settings etc.) in countries within sub-Saharan Africa. ▪ Studies conducted in clinical contexts focused on self-harm as the main presenting condition. ▪ Clinical studies concerned with self-harm as the primary condition (but not as comorbid condition, e.g., self-harm in HIV/AIDS or epilepsy).	▪ Studies focused on adolescents in prisons or borstal institutions, unless control groups in such studies allow for the evaluation of risk and protective factors of self-harm in adolescents.
Participants	▪ Studies reporting prevalence estimates of self-harm involving participants aged between 10 and 25 years. ▪ Studies reporting on the associates, risk and protective factors related to self-harm, methods of self-harm used, and reported reasons for self-harm involving participants aged 10 and 25 years with a personal self-harm history at the time of assessment for the study. ▪ Studies with wide age range but majority (90% or more) of the participants are within the age bracket of 10–25 years.	▪ Adolescents with pervasive developmental disorders, cancer, insulin-dependent diabetes, epilepsy or HIV/AIDS adolescent patients, unless control groups in such studies allow for the evaluation of risk and protective factors of self-harm. ▪ Studies involving participants within wide age ranges with the study results not disaggregated by age, making it impossible to link specific results to participants age 10–25 years, and where participants are stratified by age but with participants aged 10–25 years constituting less than 90% of the total sample which did not specifically link the reported prevalence estimates, identified risks or associates of self-harm, protective factors, methods of self-harm, or the identified reasons for self-harm to young people aged 10–25 years.
Study Designs	▪ Studies with focus on self-harm which address at least one of the four specified objectives of this review using: (1) quantitative methods (i.e., school-based, household-based, population/community-based cross-sectional survey; census; retrospective or prospective descriptive cohort designs; case controls; case reports; randomised controlled trials, and analytic cohort designs); or (2) qualitative methods (e.g., interviews, focus groups etc.); or (3) retrospective reviews of clinical records. ▪ Cross-national studies involving countries in sub-Saharan Africa and other countries outside the sub-region, which stratify and link the results to the included countries. In such instances, the specified results related to the sub-Saharan African countries were included in this review.	▪ Studies based on the same dataset reported in an earlier publication included in this review. ▪ Systematic reviews, commentaries, editorials, opinion pieces, correspondence, and articles not based on data. ▪ Where full text of the identified article was unavailable or could not be accessed. ▪ Cross-national studies involving countries in sub-Saharan Africa and other countries outside the sub-region, which did not stratify or link the results to the respective included countries.

We used EndNote (version X9.2) to collate and handle the identified records. All records were screened for eligibility by reading the titles, abstracts, methods, and results sections by EQ, with consensus discussion of 10–20% of studies with co-authors. Appraisal of the methodological quality of records used the mixed method appraisal tool (MMAT) [17]. There was substantial heterogeneity across the studies. We applied narrative synthesis to the evidence in the final set of studies; we present the prevalence estimates as median values and interquartile ranges (IQRs). We report pooled estimates (median and IQR) to aid presentation of the data, but note should be taken of the substantial heterogeneity in studies included [18, 19].

## Results

Seventy-four studies involving adolescents aged 10–25 years were included after removing duplicates - one national report on adolescent health behaviour, seven (9·4%) postgraduate theses, one book chapter and 65 (87·8%) peer-reviewed articles published in indexed academic journals (see Additional file 5).

### Characteristics of included studies

The majority of the studies, 54 (73%), used the terms “suicide attempt”, “suicidal attempt”, or “attempted suicide”, to describe self-harm. Although included studies distinguished between suicidal self-harm and non-suicidal self-harm in their findings none of the studies indicated if a definition or explanation of the core question was provided to participants [20]. Because we wanted to include all acts that meet the WHO definition, which does not include a requirement for a specific intent, given the contention about the soundness of the distinction between self-reported suicidal and non-suicidal acts [21], which is likely to be a particular problem in countries where suicide is illegal and where different languages may not readily reflect the distinction, and as individual suicide risk is known to reside in all acts of self-harm regardless of attribution, we have included all studies in our estimates of prevalence where it was clear that self-harm was the question put to participants, regardless of apparent intent.

Data were available from all four geographical sub-regions of sub-Saharan Africa. The majority (44·6%) were from five countries within Southern sub-Saharan Africa (eSwatini, Mozambique, Namibia, South Africa, and Zambia) - South Africa ranked the highest with more than half (*n* = 37) of the total included records; 30·4% were based on data from six Western sub-Saharan African countries (Benin, Ghana, Ivory Coast, Mauritania, Nigeria, and Togo); 1·1% from Congo-Brazzaville in Central sub-Saharan Africa; and 23·9% of data obtained from six countries within Eastern sub-Saharan Africa (Ethiopia, Malawi, Rwanda, Seychelles, Tanzania, and Uganda). Seventy-two of the studies were in English and two were in French.

The majority of the studies (*n* = 56; 75·6%) utilised a quantitative cross-sectional design involving questionnaires administered to participants accessed in communities/households, a charity facility, hospitals, schools and universities (see Additional file 6). Five studies included only female participants [22–26]; and one study involved only male participants [27]. The majority (*n* = 49; 66·2%) of the studies sampled students from schools and universities. Six studies (8·1%) sampled young people who were out of the school environment, including adolescents living in poor, rural, war-affected communities [28], adolescents in children’s homes [29], children and youth living in slums and streets [30, 31], adolescents living in poor urban and rural villages [32], and out-of-school youth who were unstably housed, living in poor urban neighbourhoods [33].

The total sample covered by the studies was 205,132. Thirty-nine (52·7%) of the studies used some form of random selection in recruiting their participants. Across the 74 studies, only 14 (18·9%) provided information on the size of their target population and how their sample sizes were determined.

Six (8·1%) of the studies were rated “average quality” on the MMAT, 21 studies (28·4%) were rated “above average quality”; 22 studies (29·7%) were rated “high quality”, while the remaining 25 studies (33·8%) were rated to be of “very high quality” (see Additional file 7).

### Main findings

Fifty-five (74·3%) of the studies reported prevalence estimates, though none reported lifetime, 12-month, 6-month, and 1-month estimates, with 12-month estimates favoured by the majority (*n* = 30; 54·5%). As shown in Table 2, the reported lifetime prevalence estimates ranged from 1·4% to 48·3% [28, 50]; the 12-month prevalence estimates varied between 0·9% and 35·8% [52, 72]; the reported 6-month prevalence ranged from 7·4% to 22·7% [55, 79]; and the 1-month reported prevalence estimates varied between 1·9% and 26·4% [31, 58].

**Table 2:** Prevalence Estimates of Self-harm (by year and country of publication)

Author (year) Country	Term	Setting (sample)	Prevalence estimate				Study quality
			Lifetime	12-month	6-month	1-month	
Flisher et al. (1993) [34]. South Africa.	Attempted suicide	School (7340)	–	OV = 572/7340 (7.8%)	–	–	2/5
Kebede & Ketsela (1993) [35]. Ethiopia.	Attempted suicide	School (519)	OV = 74/519 (14.3%) F = 32/232 (13.8%) M = 42/287 (14.6%)	–	–	–	5/5
Peltzer et al. (2000) [36]. South Africa.	Attempted suicide	School (366)	OV = 46/366 (12.6%)	–	–	–	3/5
Madu & Matla (2003) [37]. South Africa.	Attempted suicide	School (435)	OV = 91/435 (21%) F = 43/243 (18%) M = 48/192 (25%)	–	–	–	4/5
Wild et al. (2004) [38]. South Africa.	Suicidal attempt	School (939)	–	OV = 95/939 (10%) F = 69/519 (13.3%) M = 26/420 (6.2%)	–	–	4/5
Sommer (2005) [39]. Cross-national (South Africa & Germany)	Suicidal behaviour	School (299)	OV = 48/299 (16.1%) F = 38/185 (20.5%) M = 10/114 (8.8%)	–	–	–	2/5
Flisher et al. (2006) [40]. South Africa.	Suicidal attempt	School (10669)	–	OV = 9.1% F = 691/6066 (11.4%) M = 313/4603 (6.8%)	–	–	3/5
Shiferaw et al. (2006) [41]. Ethiopia.	Suicidal attempt	School (667)	OV = 44/667 (6.6%)	OV = 39/667 (5.8%) F = 11/155 (7.1%) M = 28/512 (5.5%)	–	–	2/5
Omigbodun et al. (2008) [20]. Nigeria.	Attempted suicide	School (1429)	–	OV = 167/1429 (11.7%) F = 87/702 (12.4%) M = 80/727 (11%)	–	–	4/5
Peltzer (2008) [42]. South Africa	Suicide attempt	School (1157)	–	OV = 278/1157 (24%)	–	–	4/5
Mashego & Madu (2009) [43]. South Africa.	Suicidal behaviour	School (142)	OV = 21/142 (14.8%) F = 14/86 (16.3%) M = 7/56 (12.5%)	–	–	–	4/5
Kinyanda et al. (2011) [28]. Uganda.	Self-injury & Suicide attempt	Community (897)	Self-injury: 13/897 (1.4%) Suicide attempt: 15/897 (1.7%)	–	–	–	5/5
Nanewortor (2011) [44]. Ghana	Attempted suicide	School (383)	OV = 31/383 (8.1%)	–	–	–	4/5
Campbell (2012) [45]. South Africa	Attempted suicide	School (1033)	OV = 129/1033 (12.5%) F = 100/552 (18.1%) M = 26/437 (5.9%)	–	–	–	3/5
Swahn et al. (2012) [30]. Uganda.	Suicidal attempt	Community (457)	–	OV = 90/457 (19.8%) F = 67/313 (21.4%) M = 23/144 (16.2%)	–	–	4/5
van Niekerk et al. (2012) [46]. South Africa.	Suicidal attempt	University (810)	OV = 47/810 (5.8%)	–	–	–	3/5
Vawda (2012) [47]. South Africa	Suicide attempt	School (219)	OV = 12/219 (5.5%)	–	–	–	3/5
Gage (2013) [25]. Ethiopia	Suicide attempt	Community (2709)	–	–	OV = 62/2709 (2.3%)	–	4/5
Muula et al. (2013) [48]. Zambia.	Self-inflicted serious injury	School (2136)	–	OV = 254/2136 (11.9%)	–	–	5/5
Shilubane et al. (2013) [49]. South Africa.	Suicidal attempt	School: 2002 SAYRBS (10549) 2008 SAYRBS (10097)	–	–	2002 SAYRBS: OV = 18.5% F = 19.5% M = 17.3% 2008 SAYRBS: OV = 21.8% F = 22.7% M = 20.8%	–	5/5
van Rooyen (2013) [50]. South Africa	Deliberate self-harm	University (603)	OV = 291/603 (48.3%)	OV = 223/6033 (7.0%)	–	–	3/5
Cheng et al. (2014) [33]. Cross-national (Nigeria, South Africa, China, India, USA).	Suicidal attempt	Community: Nigeria (449) South Africa (496)	–	Nigeria: OV = 73/449 (16.3%) F = 33/229 (14.3%) M = 40/220 (18.3%) South Africa: OV = 54/496 (10.9%) F = 22/224 (10%) M = 32/272 (11.8%)	–	–	4/5
Chinawa et al. (2014) [51]. Nigeria.	Attempted suicide	School (764)	–	OV = 96/764 (12.5%)	–	–	2/5
Lippi (2014) [52]. South Africa.	Deliberate self-harm	University (603)	OV = 278/603 (46.1%) F = 219/483 (45.3%) M = 59/120 (49.2%)	OV = 216/603 (35.8%)	–	–	3/5
Penning & Collings (2014) [53]. South Africa.	Self-injury	School (716)	–	OV = 20/716 (2.8%)	–	–	5/5
Randall et al. (2014) [54]. Benin.	Attempted suicide	School (2690)	–	OV = 761/2690 (28.3%)	–	–	5/5
Shilubane et al. (2014) [55]. South Africa.	Suicide attempt	School (591)	–	–	OV = 134/591 (22.7%) F = 52/297 (18.2%) M = 77/294 (27%)	–	4/5
Cluver et al. (2015) [56]. South Africa	Suicide attempt	Community (3401)	–	–	–	OV = 111/3401 (3.3%) F = 79/1926 (4.4%) M = 32/1475 (2.2%)	4/5
Ng et al. (2015) [57]. Rwanda	Suicidal behaviour	Community (237)	–	–	OV = 30/237 (12.6%)	–	5/5
Giru (2016) [58]. Ethiopia	Suicide attempt	School (722)	OV = 90/722 (12.5%) F = 47/336 (14%) M = 43/386 (11.1%)	–	–	OV = 14/722 (1.9%) F = 10/336 (3%) M = 4/386 (1%)	3/5
Shaikh et al. (2016) [59]. Malawi.	Suicide attempt	School (2225)	–	OV = 287/2225 (12.9%) F = 157/1188 (13.2%) M = 130/1037 (12.4%)	–	–	5/5
van der Walt (2016) [60]. South Africa	Self-harm	University (201)	OV = 39/201 (19.4%)	–	–	–	3/5
Akanni et al. (2017) [61]. Nigeria	Attempted suicide	School (300)	–	OV = 17/300 (5.7%) F = 8/135 (5.9%) M = 9/165 (5.5%)	–	–	2/5
Asante et al. (2017) [62]. Ghana	Suicide attempt	School (1973)	–	OV = 438/1973 (22.2%) F = 213/908 (23.5%) M = 225/1065 (21.1%)	–	–	5/5
Asante & Meyer-Weitz (2017) [31]. Ghana.	Suicidal attempt	Community (227)	–	–	–	OV = 60/227 (26.4%) F = 36/105 (37.5%) M = 24/122 (20.3%)	4/5
James et al. (2017) [63]. South Africa.	Suicidal attempt	School (10997)	–	–	OV = 17.8%	–	4/5
Nyandindi (2017) [64]. Tanzania	Suicide attempt	School (3793)	–	OV = 436/3793 (11.5%) F = 230/1931 (11.9%) M = 192/1862 (10.3%)	–	–	5/5
Peltzer & Pengpid (2017) [65]. Namibia	Suicide attempt	School (4531)	–	OV = 1029/4531 (22.7%) F = 604/2406 (27.4%) M = 577/2125 (24.5%)	–	–	4/5
Stansfeld et al. (2017) [66]. South Africa	Suicide attempt	School (1034)	–	OV = 139/1034 (13.4%)	–	–	4/5
Amare et al. (2018) [67]. Ethiopia	Suicide attempt	School (573)	OV = 93/573 (16.2%) F = 44/296 (14.8%) M = 49/277 (17.7%)	–	–	OV = 18/573 (3.1%)	5/5
Khuzwayo et al. (2018) [68]. South Africa	Suicide attempt	School (1687)	–	OV = 256/1687 (15.2%) F = 196/854 (22.9%) M = 60/833 (7.2%)	–	–	3/5
Liu et al. (2018) [69]. Cross-national (Benin, Ghana, Malawi, Mauritania, Namibia, & eSwatini).	Suicide attempt	School: Benin (2649) Ghana (3543) Malawi (2212) Mauritania (1976) Namibia (4410) eSwatini (3612)	–	Benin: OV = 747/2649 (28.2%) F = 260/927 (28%) M = 486/1722 (28.2%) Ghana: OV = 935/3543 (26.4%) F = 449/1637 (27.4%) M = 486/1906 (25.5%) Malawi: OV = 246/2212 (11.1%) F = 128/1175 (10.7%) M = 118/1037 (11.4%) Mauritania: OV = 334/1976 (16.9%) F = 173/1045 (16.6%) M = 160/931 (17.2%) Namibia: OV = 1129/4410 (25.6%) F = 565/2329 (24.2%) M = 564/2081 (27.1%) eSwatini: OV =585/3612 (16.2%) F = 305/1896 (16.1%) M = 280/1716 (16.3%)	–	–	5/5
van der Wal & George (2018) [70]. South Africa.	Self-harm	School (962)	OV = 167/962 (17.4%) F = 109/557 (19.4%) M = 58/405 (14.5%)	–	–	–	3/5
Baiden et al. (2019) [71]. Ghana	Suicide attempt	School (1633)	–	OV = 349/1633 (21.1%) F = 187/807 (23.2%) M = 162/826 (19.6%)	–	–	5/5
Brittain et al. (2019) [72]. South Africa	Self-harm & Attempted suicide	Community (110)	–	Self-harm: 3/110 (3%) Attempted suicide: 1/110 (0.9%)	–	–	4/5
Darré et al. (2019) [73]. Togo	Suicide attempt	School (941)	OV = 46/941 (4.9%) F = 31/753 (3.3%) M = 15/188 (1.6%)	–	–	–	3/5
Koyanagi, Oh et al. (2019) [74]. Cross-national (Benin, Ghana, Malawi, Mauritania, Mozambique, Namibia, Seychelles, Swaziland, & Tanzania).	Suicide attempt	School: Benin (1170) Ghana (1110) Malawi (2224) Mauritania (1285) Mozambique (668) Namibia (1936) Seychelles (2061) Swaziland (1318) Tanzania (2615)	–	Benin: OV = 337/1170 (28.8%) F = 104/397 (26.4%) M = 233/773 (30.1%) Ghana: OV = 295/1110 (26.6%) F = 154/565 (27.5%) M = 141/545 (25.9%) Malawi: OV = 249/2224 (11.2%) F = 115/1079 (10.7%) M = 128/1145 (11.2%) Mauritania: OV = 227/1285 (17.7%) F = 98/601 (16.3%) M = 126/684 (18.4%) Mozambique: OV = 112/668 (16.8%) F = 56/337 (16.7%) M = 57/331 (17.1%)	–	–	5/5
Koyanagi, Oh et al. (2019) [74].	Suicide attempt		–	Namibia: OV = 507/1936 (26.2%) F = 260/1106 (23.5%) M = 247/830 (29.7%) Seychelles: OV = 435/2061 (21.1%) F = 215/1041 (20.7%) M = 220/1020 (21.6%) Swaziland: OV = 202/1318 (15.3%) F = 121/803 (15.1%) M = 78/515 (15.2%) Tanzania: OV = 290/2615 (11.1%) F = 159/1391 (11.4%) M = 126/1224 (10.3%)	–	–	5/5
Koyanagi, Stubbs et al. (2019) [75]. Cross-national (including Benin, Ghana, Mauritania, Mozambique, Namibia, Seychelles, Swaziland, & Tanzania).	Suicide attempt	School: Benin (2690) Ghana (1684) Mauritania (2063) Mozambique (1918) Namibia (4531) Seychelles (2540) Swaziland (3680) Tanzania (3793)	–	Benin: 761/2690 (28.3%) Ghana: 485/1684 (28.8%) Mauritania: 359/2063 (17.4%) Mozambique: 363/1918 (18.9%) Namibia: 1178/4531 (26.0%) Seychelles: 511/2540 (20.1%) Swaziland: 607/3680 (16.5%) Tanzania: 436/3793 (11.5%)	–	–	5/5
Nguyen et al. (2019) [76]. Cross-national (Nigeria, Uganda, & Zambia).	Self-injury	Households: Nigeria (4203) Uganda (5804) Zambia (1819)	Nigeria: 123/4203 (2.9%) Uganda: 244/5804 (4.2%) Zambia: 104/1819 (5.7%)	–	–	–	5/5
Quarshie et al. (2019) [77]. Ghana	Suicide attempt	College (305)	OV = 7/305 (2.3%) F = 6/277 (2.3%) M = 1/28 (3.6%)	–	–	–	5/5
Shayo & Lawala (2019) [78]. Tanzania	Suicide attempt	School (3793)	–	OV = 422/3793 (11.1%) F = 230/1974 (11.7%) M = 192/1819 (10.6%)	–	–	5/5
Thornton et al. (2019) [79]. Cross-national (South Africa & Guyana)	Suicide attempt	Households: South Africa (175)	–	–	South Africa: 14/175 (7.4%)	–	2/5
Tolulope et al. (2019) [80]. Nigeria.	Suicide attempt	School (1015)	OV = 30/1015 (3%)	–	–	–	4/5
Uddin et al. (2019) [81]. Cross-national (Benin, Ghana, Malawi, Mauritania, Mozambique, Namibia, Swaziland, & Tanzania).	Suicide attempt	School: Benin (2579) Ghana (2195) Malawi (2191) Mauritania (1867) Mozambique (1248)	–	Benin: OV = 720/2579 (27.9%) F = 254/902 (28.2%) M = 466/1667 (28%) Ghana: OV = 607/2195 (27.7%) F = 304/1029 (29.5%) M = 294/1146 (25.7%) Malawi: OV = 251/2191 (11.5%) F = 125/1142 (11%) M = 118/1015 (11.6%)	–	–	5/5
Uddin et al. (2019) [81].	Suicide attempt	Namibia (3235) Swaziland (2547) Tanzania (2911)	–	Mauritania: OV = 322/1867 (17.3%) F = 161/983 (16.4%) M = 153/866 (17.7%) Mozambique: OV = 213/1248 (17.1%) F = 113/596 (19%) M = 95/633 (15%) Namibia: OV = 836/3235 (25.8%) F = 432/1795 (24.1%) M = 390/1409 (27.7%) Swaziland: OV = 439/2547 (17.2%) F = 245/1432 (17.1%) M = 190/1108 (17.2%) Tanzania: OV = 298/2911 (10.2%) F = 161/1448 (10.9%) M = 126/1403 (9%)	–	–	5/5
Vancampfort et al. (2019) [82]. Cross-national (Benin, Ghana, Mauritania, Mozambique, Namibia, Seychelles, & Tanzania)	Suicide attempt	School: Benin (1170) Ghana (1110) Mauritania (1285) Mozambique (668) Namibia (1936)	–	Benin: 323/1170 (27.6%) Ghana: 295/1110 (26.6%) Mauritania: 213/1285 (16.6%) Mozambique: 114/668 (16.5%) Namibia: 492/1936 (25.4%)	–	–	5/5
Vancampfort et al. (2019) [82].	Suicide attempt	Seychelles (2061) Tanzania (2615)	–	Seychelles: 423/2061 (20.5%) Tanzania: 282/2615 (10.8%)	–	–	5/5

*OV* Overall estimate

*F* Female

*M* Male

*SAYRBS* South African Youth Risk Behaviour Survey

Gage’s (2013) [25] reported prevalence period was 3 months. Van Rooyen’s (2013) [50] reported prevalence period was 11 months

The majority of the 55 prevalence studies (*n* = 46; 83·6%) focused on suicidal self-harm, seven studies (12·7%) focused on non-suicidal self-harm [48, 50, 52, 53, 60, 70, 76], while two studies simultaneously reported the prevalence estimates of both suicidal self-harm (suicidal attempt) and non-suicidal self-harm (self-injury) [28, 72].

**Fig. 1:**
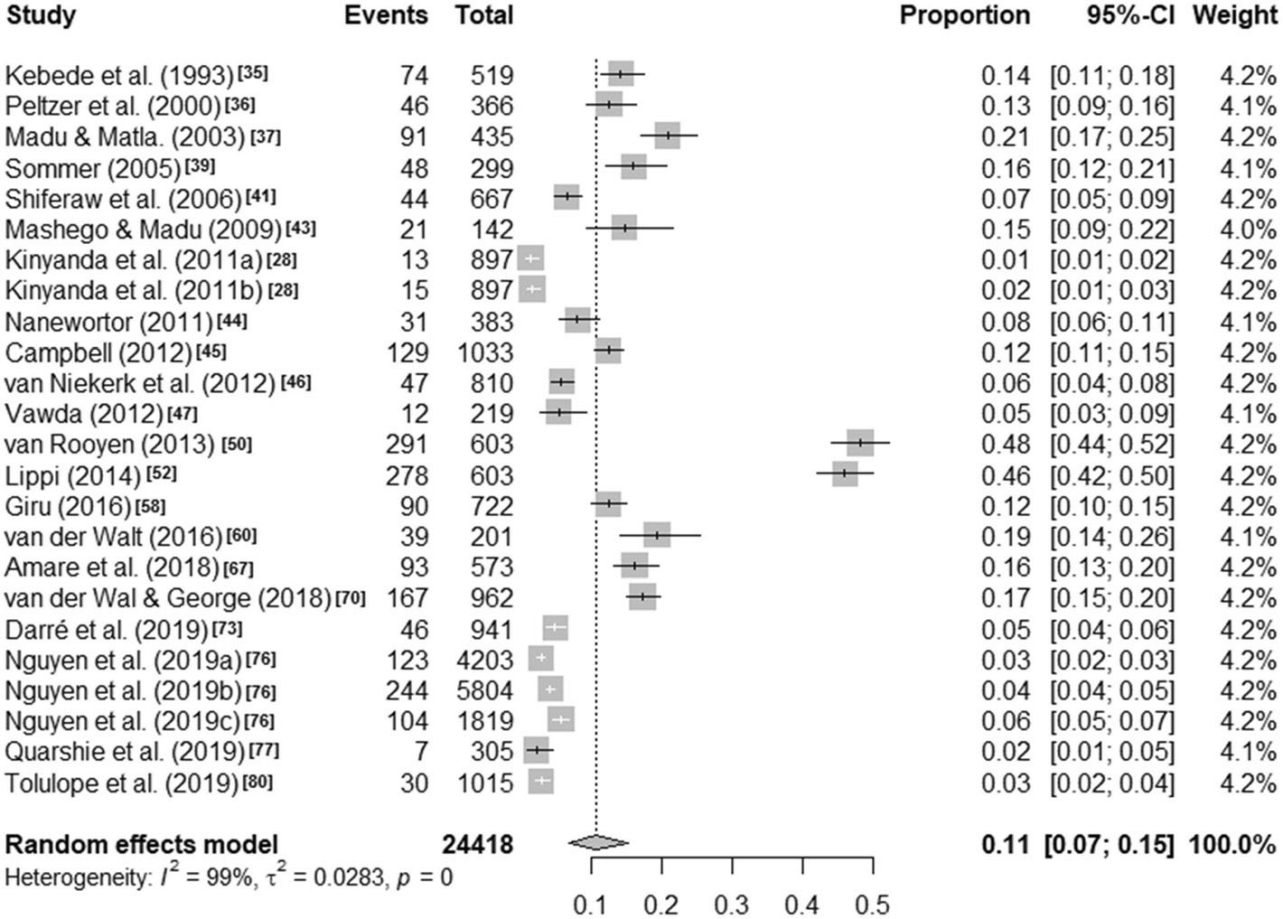
Lifetime prevalence estimates of self-harm

**Fig. 2:**
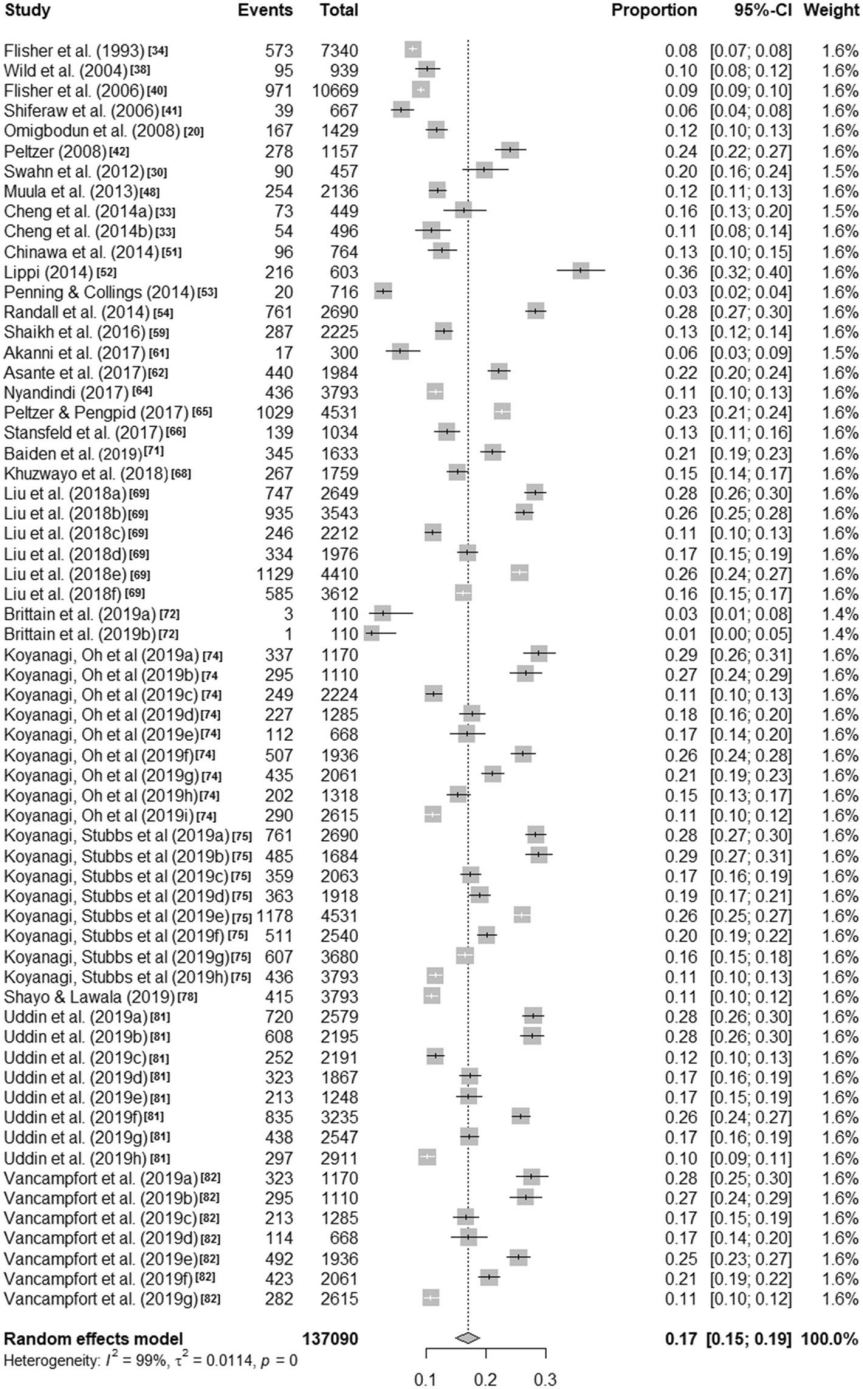
12-month prevalence estimates of self-harm

**Fig. 3:**
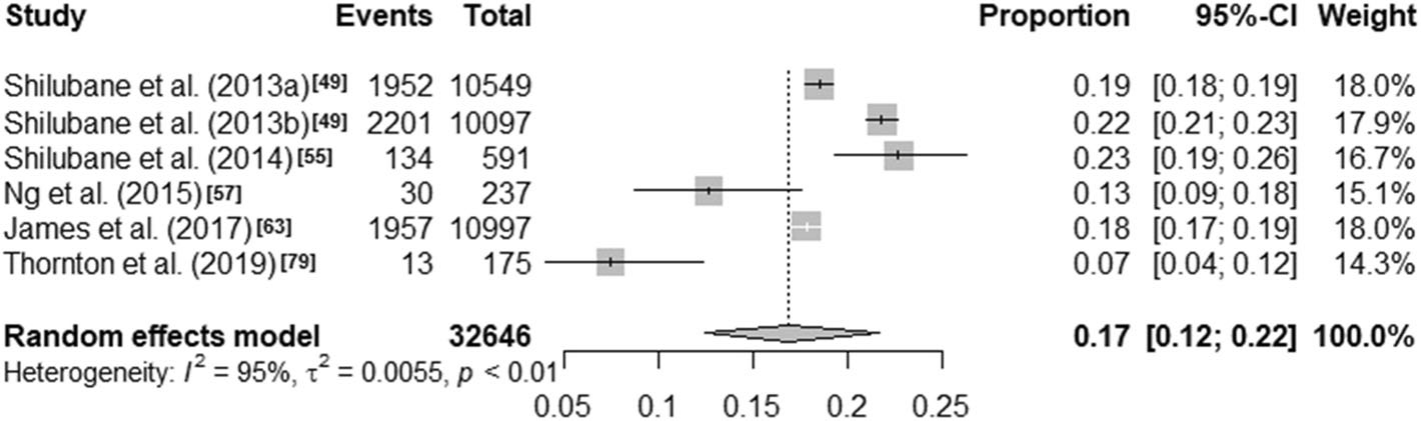
6-month prevalence estimates of self-harm

**Fig. 4:**
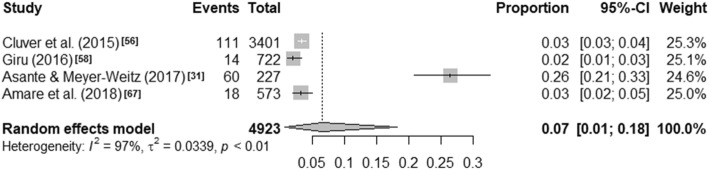
1-month prevalence estimates of self-harm

**Fig. 5:**
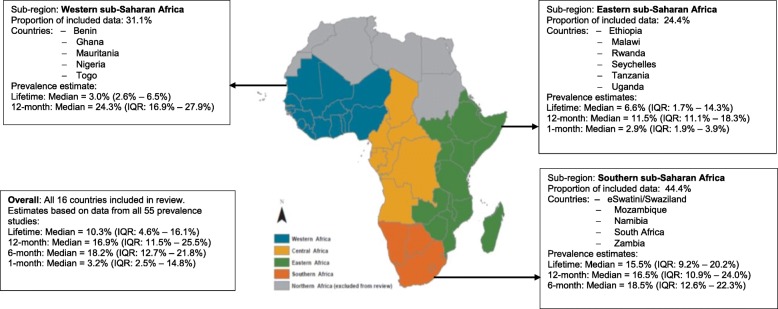
Median and interquartile range (IQR) of prevalence estimates of adolescent self-harm in sub-Saharan Africa. Source: Map created by authors, based on the list of countries within the sub-regional division of sub-Saharan Africa by the United Nations Statistics Division’s classification (list accessed on January 20, 2019: https://unstats.un.org/unsd/methodology/m49/)

Visual inspection of the forest plots and the I^2^ value ranges (98·84% to 99·71%, p < ·001) indicate that heterogeneity across each of these summaries is substantial (see Figs. 1, 2, 3 and 4). Median values with interquartile ranges were computed for the overall and sub-regional reported prevalence estimates (see Fig. 5). Considerable variability was found across the ranges of prevalence estimates reported: the median lifetime prevalence estimate was 10·3% (interquartile range [IQR] of 4·6% – 16·1%) and the median 12-month prevalence estimate was 16·9% (IQR: 11·5% – 25·5%). Studies from Western sub-Saharan Africa reported the highest 12-month prevalence estimates (median = 24·3%; IQR = 16·9% – 27·9%), while studies from Eastern (median = 11·5%; IQR = 11·1% – 18·3%) and Southern (median = 16·5%; IQR = 10·9% – 24·0%) sub-Saharan Africa reported relatively similar median 12-month prevalence estimates.

Twenty (27%) of the 74 studies reported on the methods of self-harm (Table 3). Overdose of medication was frequently reported from clinic-based studies, while self-cutting was the predominant method reported in the non-clinic based studies.

**Table 3:** Predominant Form / Method of Self-harm by year of publication

Author (year) Country	Setting	Sample (Sex)	Reported method of self-harm		Study quality
			Self-Poisoning	Self-Injury	
Cummins & Allwood (1984) [83] South Africa	General hospital	*N* = 81 (F = 54, M = 27)	▪ Overdose = 64/81 (79%) F = 46/54 (85.2%) M = 18/27 (66.7%)	–	3/5
Schlebusch (1985) [84] South Africa	General hospital	*N* = 159 (F = 115, M = 44)	▪ Overdose, 151/159 (95%) F = 112/115 (97.4%) M = 39/44 (88.6%)	▪ Wrist/arm cutting = 8/159 (5%) F = 5/115 (4.3%) M = 3/44 (6.8%)	4/5
Pillay (1987) [85] South Africa	General hospital	*N* = 55 (F = 42, M = 13)	▪ Overdose = 55/55 (100%)	–	3/5
Pillay (1988) [86] South Africa	General hospital	*N* = 87 (F = 68, M = 19)	▪ Self-poisoning = 81/87 (93.1%) F = 67/68 (98.5%) M = 14/19 (73.7%) ▪ Carbon monoxide = 1/87 (1.1%) F = 0/68 M = 1/19 (5.3%)	▪ Wrist cutting = 1/87 (1.1%) F = 1/68 (1.5%) M = 0/19 ▪ Hanging = 2/87 (2.3%) F = 0/68 M = 2/19 (10.5%) ▪ Stabbing = 1/87 (1.1%) F = 0/68 M = 1/19 (5.3%) ▪ Jumping from height = 1/87 (1.1%) F = 0/68 M = 1/19 (5.3%)	3/5
Pillay & Wassenaar (1991) [87] South Africa	General hospital	*N* = 40 (F = 26, M = 14)	Ingestion of: ▪ Medicines = 38/40 (95%) ▪ Pesticides = 2/40 (5%)	–	3/5
Mhlongo & Peltzer (1999) [88] South Africa	General hospital	*N* = 100 (F = 63, M = 37)	▪ Paraffin = 36/100 (36%) ▪ Methylated spirit = 12/100 (12%) ▪ Shampoo = 11/100 (11%) ▪ Pesticides = 10/100 (10%) ▪ Detergent = 9/100 (9%) ▪ Battery acid = 6/100 (6%) ▪ Medicaments = 3/100 (3%) ▪ Ingestion of glass = 4/100 (4%)	Hanging = 9/100 (9%)	3/5
Madu & Matla (2003) [37] South Africa	School	*N* = 435 (F = 243, M = 192)	▪ Self-poisoning = 40/435 (9.2%) F = 21/243 (8.6%) M = 19/192 (9.9%) ▪ Drug overdose = 23/435 (5.3%) F = 13/243 (5.3%) M = 10/192 (5.2)	▪ Hanging = 20/435 (4.6%) F = 5/243 (2.1%) M = 15/192 (7.8%) ▪ Stabbing = 2/435 (0.5%) F = 0/243 M = 2/192 (1%)	4/5
Sommer (2005) [39] South Africa	School	*N* = 299 (F = 185, M = 114)	▪ Overdose = 141/299 (47.2%)	▪ Wrist cutting = 133/299 (44.4%) ▪ Jumping from height = 25/299 (8.4%)	2/5
Yéo-Tenena et al. (2010) [89] Ivory Coast	Hospital	*N* = 42 (F = 33, M = 9)	▪ Chloroquine = 26/42 (61.9%) ▪ Psychotropic = 8/42 (19%) ▪ Paracetamol = 4/42 (9.5%) ▪ Metronidazole = 3/42 (7.1%) ▪ Muriatic acid = 3/42 (7.1%) ▪ Sodium hypochlorite = 2/42 (4.8%) ▪ Ethyl alcohol = 2/42 (4.8%) ▪ Rat poison = 2/42 (4.8%) ▪ Thinner = 1/42 (2.4%)	▪ Hanging = 1/42 (2.4%)	4/5
Beekrum et al. (2011) [24] South Africa	Hospital	N = 10 (F = 10)	▪ All participants took overdose of prescription medication belonging to a family member: benzodiazepines, steroidal anti-inflammatories, and various blood pressure medications.		5/5
Okoko et al. (2011) [90] Congo Brazzaville	Hospital	*N* = 62 (F = 50, M = 12)	▪ Overdose = 53 (85.5%) ▪ Caustic soda = 6 (9.7%) ▪ Powder = 1 (1.6%) ▪ Rat poison = 1 (1.6%)	▪ Hanging = 1 (1.6%)	4/5
Pretorius (2011) [29] South Africa	Children’s homes	*N* = 12 (F = 10, M = 2)	–	▪ Cutting = 11 (91.6%) ▪ Carving words into skin = 11 (91.6%) ▪ Broken own bones = 9 (75%) ▪ Punching self = 8 (66.6%) ▪ Sharp objects through skin = 8 (66.6) ▪ Burning with a lighter or match = 7 (58.3%) ▪ Carving pictures or patterns into skin = 7 (58.3%) ▪ Scratching = 4 (33.3%) ▪ Rubbing glass into skin = 4 (33.3%) ▪ Banging of head = 4 (33.3%) ▪ Preventing wounds from healing = 4 (33.3%) ▪ Burning with a cigarette = 3 (25%) ▪ Biting = 1 (8.3%) ▪ Dripping acid onto skin = 1 (8.3%) ▪ Bleach or oven cleaner onto skin = 1 (8.3%) ▪ Rubbing sandpaper = 2 (16.6%).	3/5
Fine et al. (2012) [91] South Africa	Hospital	*N* = 50 (F = 31 M = 19)	▪ Overdose = 17/50 (34%) ▪ Poisoning = 2/50 (4%) ▪ Drowning = 2/50 (4%)	▪ Cutting = 36/50 (75%) ▪ Hanging = 10 (20%) ▪ Jumping from a height = 3/50 (6%)	3/5
Shilubane et al. (2012) [32] South Africa	Community	*N* = 14 (F = 8, M = 6)	Ingestion of: ▪ Medications = 9/14 (64.3%) ▪ Paraffin = 1/14 (7.1%) ▪ Disinfectant = 1/14 (7.1)	▪ Burning = 1/14 (7.1%) ▪ Hanging = 1/14 (7.1%)	4/5
Van Rooyen (2013)^a^ [50] Lippi (2014)^a^ [52] South Africa	University	*N* = 603 (F = 483, M = 120)	–	▪ Cutting = 132/603 (21.9%) ▪ Severe scratching = 93/603 (15.4%) ▪ Carving words into skin = 70/603 (11.6%) ▪ Burning with lighter or match = 66/603 (10.9%) ▪ Sticking objects into skin = 52/603 (8.6%) ▪ Punching self = 48/603 (8%) ▪ Carving pictures into skin = 44/603 (7.3%) ▪ Burning with cigarette = 42/603 (7%) ▪ Interfering with wound healing = 36/603 (6.5%) ▪ Banging head = 30/603 (5%)	3/5
van der Walt (2016) [60] South Africa	University	*N* = 201 (F = 110, M = 91)	▪ Alcohol abuse = 46/201 (22.9%) ▪ Overdose = 25/201 (12.4%) ▪ Medication abuse = 12/201 (6%)	▪ Hitting self = 26/201 (12.9%) ▪ Head banging = 24/201 (11.9%) ▪ Cutting = 18/201 (9%) ▪ Scratching = 17/201 (8.5%) ▪ Exercised an injury on purpose = 12/201 (6%) ▪ Prevented wounds from healing = 10/201 (5%) ▪ Burning = 4/201 (2%) ▪ Reckless driving = 21/201 (10.4%)	3/5
Meissner & Bantjes (2017) [27] South Africa	University	N = 4 (M = 4)	–	▪ Hanging = 2/4 (50%) ▪ Car accident = 2/4 (50%)	5/5
Kritzinger (2018) [26] South Africa	Hospital	N = 10 (F = 10)	▪ Overdose ▪ Rat poison ▪ Furniture oil	–	5/5

*F* Female

*M* Male

^a^ The studies by Lippi (2014) [52] and van Rooyen (2013) [50] were based on the same dataset, the 2009 University of Pretoria student survey in South Africa

Overall, 48 (64·9%) of the 74 studies reported on the associates, risks and protective factors. The evidence was organised by reported associates into four main domains: personal, family, school, and interpersonal (non-family). The interpersonal (non-family) included circumstances related to the individuals’ relationships with peers and neighbours, and other social relationships and interactions outside the family and school contexts. Additionally, based on the strong association of abuse and violence victimisation (within and outside the family context) and self-harm [11, 92–94], we created a separate category, *abuse and violence*, to capture all factors related to psychological, physical, emotional, and sexual abuse items.

Results are shown in Table 4. Common examples of associations included, at the personal level, depression, hopelessness and psychiatric illness; at the family level, conflict with parents, parental divorce; at school level, academic failure, and for the interpersonal level, relationship breakups and problems, and lack of social support. Abuse and violence-related factors included sexual abuse, dating violence, bullying, and physical fights. Only one study reported risk factors related to self-harm [56], while no study reported protective factors against self-harm.

**Table 4:** Associates, Risk and Protective Factors of Self-Harm (by year of publication)

Author (year) Country	Associates / Risk Factors					Study quality
	Personal ^a^	Family ^b^	School ^c^	Interpersonal ^d^	Abuse and violence ^e^	
Cummins & Allwood (1984) [83] South Africa	▪ Psychiatric disturbance	▪ Family dysfunction (including divorce) ▪ Family psychiatric illness	▪ School problems	▪ Socialisation problems	–	3/5
Pillay (1987) [85] South Africa	▪ Medical/psychiatric illness	▪ Problems with Parents ▪ Problems with siblings ▪ Marital problems	▪ School problems	▪ Problems with boyfriend or girlfriend	–	3/5
Sefa-Dedeh & Canetto (1992) [22] Ghana	–	▪ Family harassment and dispute ▪ Failed sense of autonomy in the family	–	–	–	4/5
Kebede & Ketsela (1993) [35] Ethiopia	▪ Hopelessness ▪ Heavy alcohol intake	–	▪ Lower school grade	–	–	5/5
Pillay & Wassenaar (1997) [95] South Africa	▪ Depression	▪ Lower family adaptability ▪ Low family cohesion ▪ Low family satisfaction ▪ Hopelessness ▪ Psychiatric disturbances.	▪ Problems at school	▪ Romantic relationship problems	–	5/5
Wassenaar et al. (1998) [23] South Africa	▪ Hopelessness	▪ Family communication breakdown ▪ Conflict with parents ▪ Authoritarian patriarchy.	–	–	–	4/5
Mhlongo & Peltzer (1999) [88] South Africa	▪ AIDS phobia ▪ Teenage pregnancy ▪ Mental illness ▪ Unemployment ▪ Financial problems	▪ Problem with parents	▪ Academic failure	▪ Romantic relationship problems	–	3/5
Peltzer et al. (2000) [36] South Africa	▪ Suicidal ideation ▪ Suicide intent	▪ History of family suicide ▪ Parental divorced ▪ Large family size	–	▪ History of suicide by friend	–	3/5
Madu & Matla (2004) [96] South Africa	–	▪ Family conflict	–	–	–	4/5
Wild et al. (2004) [38] South Africa	▪ Depression ▪ Poor global self-worth ▪ Poor body image ▪ Female sex	–	▪ Poor schoolwork	▪ Problems with peers	–	4/5
Sommer (2005) [39] South Africa	▪ Female sex ▪ Previous psychiatric contact	▪ Perceived lack of family support ▪ Suicide attempt in the family	–	▪ Death of a friend	–	2/5
Shiferaw et al. (2006) [41] Ethiopia	▪ Being sexually active ▪ Female sex ▪ Unwanted pregnancy ▪ Boredom ▪ HIV/AIDS positive status	▪ Family member attempted suicide ▪ Lack of family support ▪ Living with both biological parents	▪ Academic under-achievement	▪ Friend suicide attempt ▪ Romantic relationship problems	–	2/5
Omigbodun et al. (2008) [20] Nigeria	▪ Drinking alcohol ▪ Having to go hungry	▪ Unstable family life ▪ Having a mother who had been married more than once ▪ Living in urban location	–	–	▪ Sexual abuse ▪ Physical attack ▪ Physical fights	4/5
Yéo-Tenena et al. (2010) [89] Ivory Coast	▪ Psychiatric problems (depression, substance addiction) ▪ Previous suicide attempt ▪ Emotional problems	▪ Familial conflict	▪ School failure	▪ Unwanted pregnancy	▪ Sexual abuse	4/5
Beekrum et al. (2011) [24] South Africa	▪ Hopelessness and despair	▪ Previous suicide or attempted suicide by a close family member ▪ Conflictual, disengaged or over-protective family relationships ▪ Strained adolescent-parent communication ▪ Conflicting social roles and values in the context of contemporary acculturation pressures	▪ Academic failure	▪ Breakup ▪ Lack of social support	▪ Physical and emotional abuse in the family	5/5
Okoko et al. (2011) [90] The Congo	▪ Previous suicide attempt ▪ Psychosis ▪ Alcohol abuse ▪ Drugs abuse ▪ Emotional breakdown	▪ Conflict with parents ▪ Difficulty with family communication ▪ Parental divorce ▪ Parental death ▪ Kinship fostering ▪ Living in a stepfamily	▪ School problems	▪ Breakup	▪ Domestic violence victimisation ▪ Sexual abuse ▪ Neglect ▪ Incest	4/5
Pretorius (2011) [29] South Africa	▪ Personal history of suicide attempts suicide; ▪ Previous diagnosis of mood disorders (i.e., major depression, and bipolar disorder)	▪ Experience of human trafficking before removal from parental care ▪ Dysfunctional parenting (unavailability, conflict, or alcoholism) before removal from parental care ▪ Family history of attempted suicide	–	▪ Observation of the self-harm of another adolescent at the same children’s home	▪ Abuse (i.e., physical, sexual, and emotional abuse) before removal from parental care.	3/5
Campbell (2012) [45] South Africa	▪ Female sex; ▪ Coloured race;	▪ Stressful relationships with parents and extended family ▪ Financial hardship	–	▪ Stressful romantic relationship ▪ Negative life events	–	3/5
Shilubane et al. (2012) [32] South Africa	▪ Perceived accusations of negative behaviour ▪ Feelings of physical rejection ▪ Acute negative mood (e.g., depression, anger, hopelessness) ▪ Being unaware of community-support resources ▪ Personal history of attempted suicide	▪ Conflictual and strained family relationships ▪ Lack of family support ▪ Family member HIV positive status ▪ Death of close family member ▪ Family history of attempted suicide ▪ Family poverty	–	▪ Lack of trusted peer support ▪ Peer suicide attempt	–	4/5
Swahn et al. (2012) [30] Uganda	▪ Sadness ▪ Expectations of dying prior to age 30	▪ Parental neglect due to alcohol use	–	–	–	4/5
Vawda (2012) [47] South Africa	–	▪ Family member suicide	–	–	–	3/5
Gage (2013) [25] Ethiopia	▪ Currently employed ▪ Lost much sleep over worry ▪ Depression	▪ Receiving marriage request ▪ Both parents deceased	–	▪ Community involvement in child marriage prevention	▪ Sexual violence victimisation	4/5
Muula et al. (2013) [48] Zambia	▪ Female sex ▪ Aged ≤14 yrs. ▪ Loneliness ▪ Sleeplessness due to worry ▪ Hopelessness ▪ Suicidal ideation ▪ Marijuana use ▪ Drunkenness	–	–	–	▪ Use of dagga	5/5
Shilubane et al. (2013) [49] South Africa	▪ Female sex ▪ Hopelessness ▪ Feeling unsafe ▪ Substance use ▪ Having unsafe sex ▪ Older adolescence ▪ Body dissatisfaction.	–	▪ Lower grade	–	▪ Violence	5/5
Chinawa et al. (2014) [51] Nigeria	▪ Depression ▪ Alcohol and drug use	–	–	–	–	2/5
Penning & Collings (2014) [53] South Africa	▪ Female sex	▪ Domestic injury	–	–	▪ Domestic assault ▪ Rape ▪ Emotional abuse ▪ Negative child sexual abuse appraisals	5/5
Randall et al. (2014) [54] Benin	▪ Male sex ▪ Anxiety ▪ Loneliness ▪ Substance use	–	–	–	▪ Being attacked	5/5
Lippi (2014) [52] South Africa	▪ Severe depression	–	–	–		3/5
Cluver et al. (2015) [56] South Africa	▪ Older adolescence ▪ Female sex ▪ Orphanhood by AIDS, ▪ Previous suicide attempt	▪ Parental AIDS-illness ▪ Food insecurity	–	–	▪ Severe physical abuse ▪ Severe emotional abuse ▪ Sexual abuse or rape ▪ Community violence ▪ Domestic violence ▪ Orphanhood by homicide	4/5
Ng et al. (2015) [57] Rwanda	▪ Child mental health symptoms (i.e., Depression above diagnostic threshold; conduct problems).	▪ Parenting style	–	–	–	5/5
Giru (2016) [58] Ethiopia	▪ Family history of suicide ▪ Loneliness ▪ Hopelessness ▪ Mental illness ▪ Financial loss	▪ Family conflict ▪ Death in family	▪ Academic failure	▪ Lack of social support	–	3/5
Shaikh et al. (2016) [59] Malawi	▪ Female sex ▪ Early sexual debut ▪ Serious injury ▪ Loneliness ▪ Anxiety ▪ Suicide ideation ▪ Suicide planning ▪ Alcohol use	▪ Parental tobacco use	–	▪ Lifetime sexual partners ▪ Number of days people smoked in presence weekly ▪ Having many close friends	▪ Bullied ▪ Physical fight ▪ Physically attacked ▪ Physically bullied	5/5
Asante et al. (2017) [62] Ghana	▪ Anxiety ▪ Loneliness	▪ Parental understanding	–	▪ Food insecurity ▪ Having many close friends	▪ Bullied ▪ Being attacked ▪ Fighting	5/5
Asante & Meyer-Weitz (2017) [31] Ghana	▪ Female sex ▪ Aged 15 years or older ▪ Smoking ▪ Past alcohol use ▪ Present alcohol use ▪ Marijuana use ▪ Survival sex	–	–	–	▪ Assaulted with a weapon ▪ Having been robbed	4/5
Peltzer & Pengpid (2017) [65] Namibia	▪ Health risk behaviours ▪ Hunger ▪ Parental support	–	–	–	–	4/5
Amare et al. (2018) [67] Ethiopia	▪ Living alone ▪ Loneliness ▪ Hopelessness ▪ Sleep disturbance worries ▪ Being physically hurt	–	▪ Truancy	▪ Poor social support	–	5/5
Khuzwayo et al. (2018) [68] South Africa	▪ Aged 16 years and above ▪ Female sex ▪ Cannabis use	–	–	–	▪ Threatened in school with a weapon ▪ Bullied in school ▪ Dating violence victimisation ▪ Cyber bullying	3/5
Kritzinger (2018) [26] South Africa	▪ Anger ▪ Low mood ▪ Suicidal ideation ▪ Previous suicide attempt ▪ Impulsivity ▪ Unemployment	▪ Conflict with parents	–	▪ Breakup ▪ Loss of significant other	▪ Domestic abuse victimisation	5/5
van der Wal & George (2018) [70] South Africa	▪ Emotional reactivity ▪ Tension-reduction coping	–	–	▪ Social support	–	3/5
Baiden et al. (2019) [71] Ghana	▪ Anxiety ▪ Illicit substance use ▪ Physical activity	–	–	▪ Having a close friend	▪ Bullying victimisation	5/5
Carvalho et al. (2019) [97] Cross-national study (Benin, Ghana, Mozambique, Namibia, & Tanzania).	▪ Cannabis use	–	–	–	–	5/5
Darré et al. (2019) [73] Togo	▪ Female sex ▪ Being aged > 18 ▪ Sentimental problems ▪ Health problems ▪ Loneliness ▪ Unwanted pregnancy ▪ Distaste of life ▪ Abstinence	▪ Family history of suicide ▪ Financial problems ▪ Family problems ▪ Absence of parents	–	▪ Living as a couple ▪ Death of a loved one	–	3/5
Koyanagi, Oh et al. (2019) [74] Cross-national study (Benin, Ghana, Malawi, Mauritania, Mozambique, Namibia, Seychelles, Swaziland, & Tanzania).	–	–	–	–	▪ Bullying victimisation	5/5
Koyanagi, Stubbs, et al. (2019) [75] Cross-national (Benin, Ghana, Mauritania, Mozambique, Namibia, Seychelles, Swaziland, & Tanzania).	–	▪ Children and adolescent food insecurity	–	–	–	5/5
Nguyen et al. (2019) [76] Cross-national (Nigeria, Uganda, & Zambia).	–	▪ Orphanhood prior to age 18	–	–	▪ Coerced/forced sexual initiation	5/5
Shayo & Lawala (2019) [78] Tanzania	▪ Loneliness ▪ Anxiety ▪ Younger age	▪ Food insecurity ▪ Parental care	–	–	–	5/5
Thornton et al. (2019) [79] Cross-national (South Africa & Guyana)	–	–	–	▪ Social stress	–	2/5
Vancampfort et al. (2019) [82] Cross-national (Benin, Ghana, Mauritania, Mozambique, Namibia, Seychelles, & Tanzania)	▪ Sedentary leisure-time	–	–	–	–	5/5

^a^Personal level factors: These include personal characteristics and histories, and factors related to personal (mental) health conditions

^b^Family level factors: These cover factors and circumstances within the family, and relationships and interactions with family members

^c^School-level factors: These relate to academic performance and relationships and circumstances within the school context

^d^Interpersonal level factors: These are circumstances related to the individual’s relationships with peers and neighbours, and other social relationships and interactions outside the family and school contexts

^e^Abuse and violence: Based on previous evidence, we created this category to include all abuse and violence items – that is psychological, physical, emotional, and sexual abuse victimisation items

Because of the substantial heterogeneity in samples, definition and measurement of associations, we regard any attempt at comparison or pooling of the reported prevalences of these associations (as opposed to simply their presence or absence) as potentially misleading. We noted, however, the proportion of included studies that reported associations in each category as follows: Personal = 41 / 48; Family = 31 / 48; Interpersonal = 24 / 48; Abuse and violence = 19 / 48; School = 13 / 48.

We further categorised the self-reported reasons for self-harm into “intrapersonal” (i.e., reasons intended to change one’s state or circumstances), and “interpersonal” (i.e., reasons intended to change the state or circumstances of significant others). Eight (10·8%) studies included self-reported reasons for self-harm – see Table 5. Five of these were clinic-based, [22–24, 26, 88] while three were non-clinic based [27, 29, 50].

**Table 5:** Reported Reasons for Self-harm (by year of publication)

Author (year) Country	Term	Setting. Design. (Sample).	Reported Reasons (n [%])		Study quality
			Intrapersonal Reasons ^a^	Interpersonal Reasons ^b^	
Sefa-Dedeh & Canetto (1992) [22] Ghana	Attempted suicide	General Hospital. Qualitative clinical case study of clinical records. (Two cases: Only Case A included in review).	To: ▪ Die ▪ Validate self	To: ▪ Get revenge against parents ▪ Make parents feel guilty; ▪ Obtain empathy and understanding from family. ▪ Regain control over social relationships and resources.	4/5
Wassenaar et al. (1998) [23] South Africa	Attempted suicide	General Hospital. Qualitative clinical case study of clinical records. (Three cases: Only Case 2 included in review).	To die	To resolve conflict with parents.	4/5
Mhlongo & Peltzer (1999) [88] South Africa	Parasuicide	General hospital. Patients’ records and interviews with patients presenting with self-harm. (n = 100)	To die (27 [27%])	To demonstrate, usually, against family conflicts and abuse (58 [58%])	3/5
Beekrum et al. (2011) [24] South Africa	Non-fatal suicidal behaviour	General hospital. Qualitative case study. (*n* = 10)	To: ^c^ ▪ Stop feelings of hopelessness and despair. ▪ Get rid of negative thoughts.	To: ^c^ ▪ Let others (e.g., boyfriend, or parent) change their behaviour or attitudes. ▪ Communicate distress related to conflict with parents, parental conflict, high parental expectations, and peer-cultural conflict. ▪ Get parents/family to understand their problems.	5/5
Pretorius (2011) [29] South Africa	Deliberate self-harm	Children’s homes. Mixed methods approach. (n = 12)	To: ▪ Stop bad feelings (8 [66.6%]) ▪ Feel relaxed (7 [58.3%]) ▪ Feel something, even if it was pain (7 [58.3%]) ▪ Punish self (5 [41.6%]) ▪ Relieve feeling ‘numb’/empty (5 [41.6%])	To: ▪ Get control of a situation (5 [41.6%]) ▪ Receive more attention from guardians /caregivers/ friends (2 [16.6%]) ▪ Get guardians/caregivers to understand you (2 [16.6%]) ▪ Get help (1 [8.3%])	3/5
van Rooyen (2013) [50] South Africa	Deliberate self-harm	University. Cross-sectional survey of students. (n = 603)	To: ^c^ ▪ Stop bad feeling ▪ Relieve feeling numb or empty ▪ Punish yourself ▪ Feel relaxed ▪ Get control of a situation ▪ Feel part of a group ▪ Be like someone you respect ▪ Avoid having to do something unpleasant you don’t want to do	To: ^c^ ▪ Let others know how desperate you were ▪ Try to get a reaction from someone, even if it’s a negative reaction ▪ Receive more attention from your parents or friends ▪ Get your parents to understand or notice you ▪ Get other people to act differently or change ▪ Avoid school, work, or other activities ▪ Avoid being with people	3/5
Meissner & Bantjes (2017) [27] South Africa	Attempted suicide	University. One-to-one semi-structured qualitative interviews with students with histories of attempted suicide. (n = 4)	To: ^c^ ▪ Escape feeling trapped ▪ Avoid suicide ▪ Distract from painful memories ▪ Die	To: ^c^ ▪ Make emotional pain visible to others ▪ Disconnect from others	5/5
Kritzinger (2018) [26] South Africa	Non-Fatal Suicidal Behaviour	General hospital. Qualitative case study approach: One-to-one semi-structured interviews with clinical sample of adolescents. (n = 10)	To: ^c^ ▪ Escape unbearable thoughts ▪ End sense of meaninglessness ▪ Die	To: ^c^ ▪ Escape a painful/unbearable situation ▪ Make parents change their mind/behaviour.	5/5

^a^ Intrapersonal reasons (i.e., reasons intended to change one’s state or circumstances): reasons or motives relate to desired changes in one’s personal or internal state, including changes in sensations, emotional states or thoughts

^b^ Interpersonal reasons (i.e., reasons intended to change the state or circumstances of significant others): include desired changes within one’s social environment, such as communicating distress to someone, or to influence the behaviour of others or to punish others

^c^ Frequency distribution of reasons not reported

Across the eight studies reporting reasons for self-harm, participants concurrently reported both intrapersonal and interpersonal reasons for engaging in self-harm with no clear pattern discernible.

The findings of the prevalence studies regarding associates of self-harm were mixed in terms of age, although more reported higher estimates among young people between the ages of 15 and 17 years, compared to those aged 14 years and below, and 18 years or above [35, 39, 43, 73].

The majority of the prevalence studies reported higher estimates among female adolescents than in male adolescents [30, 34, 40, 45, 58, 59, 61, 62, 65, 73, 78], although seven studies (12·7%) found higher prevalence estimates in male adolescents [33, 35, 37, 52, 55, 67, 77].

## Discussion

There are clearly problems with the literature we found. Despite the number of reported studies, and the extended search, we identified research from fewer than 40% (18/46) of the countries across the sub-region, and half of all studies came from one country – South Africa. This represents a serious gap in our knowledge about population mental health in those countries from which research is missing.

The reported prevalence estimates showed considerable variations within and between the countries and sub-regions of sub-Saharan Africa. Undoubtedly, real variations are likely to exist but there must also be methodological reasons to explain, for example, that estimates of lifetime prevalence appear lower than 12-month prevalence. One explanation is the origin and contextual relevance of the measures used by the studies, and the lack of consistency in definitions and choice of measures across studies. In this respect it is worth noting the widespread use of the term “attempted suicide” in studies which did not explain the term to those responding to the question. The consequence is that studies cannot be reliably categorised as reporting the prevalence of suicidal or non-suicidal behaviour. For example – as shown on Table 5 respondents in a study about “attempted suicide” might report that they intended to die as the result of an act while others might report that the act was intended to prevent suicide, or that the act was designed to change the nature of their relationships with others. We found no substantial study where participants were asked in detail about reasons for self-harm, after responding with an initial affirmative to a question about attempted suicide. Respondent bias due to the illegality, sensitivity, stigma, and taboo against suicide and other self-destructive behaviours in Africa [98] is likely to vary according to how and by whom inquiries were made.

Even so, the median 12-month prevalence estimate of 16·9% (IQR: 11·5% – 25·5%), and median 6-month prevalence estimate of 18·2% (IQR: 12·7% – 21·8%) particularly had reasonably narrow confidence intervals. What is striking is that these figures are of the same order as those reported from Europe and North America, as is the finding that young women report more self-harm than young men. Similarly, methods of self-harm were similar with overdose of medication frequently reported in clinic-based studies, while self-cutting was the predominant method reported in the non-clinic based studies.

Generally, the studies reported multiple factors to be associated with self-harm at the personal level - sex, age, depression, hopelessness, psychiatric illness, alcohol and illicit drug use, at the family level - conflict with parents, at school-level - academic failure, bullying victimisation, truancy, and at the interpersonal level - breakup, sexual and physical abuse, romantic relationship problems, social support.

Various forms of abuse and violence victimisation occurring in the family, school, and interpersonal contexts were also reported. It was not possible to tell from all the studies we reviewed how often relationship problems in family, school, or social groups was marked by violence or abuse, but the circumstances of life in Ghana suggests the possibility of frequent exposure to such experiences. Relative to high-income countries, these circumstances are arguably likely to be more common in sub-Saharan Africa due to poverty, unemployment, death of parents (to AIDS), physical and sexual abuses – including (forced) child marriage – displacement by wars and conflicts, substance use and abuse (due to less than ideally regulated access to prescription medication), family conflict, among others [99, 100]. Under the circumstances, it is surprising that rates are not higher in African studies.

Findings from the qualitative studies in this review suggest some reasons for the high levels of self-harm among young people: entrenched cultural and family rules of comportment and norms of obedience and respect and the sense of powerlessness experienced by both boys and girls as linked to self-harm [22–24, 27, 32]. The higher prevalence estimates in females have been attributed to socially and religiously sanctioned oppression and exploitative normative gender role discrimination against women and girls [101]. Compared to young males, young females tend to be victims of more domestic chore burdens, caretaking responsibilities, sexual abuse and exploitation, exclusion from education, unemployment, and exclusion from decision making [11, 101]. Thus, as found in regional reviews of studies involving adult samples [11, 12] and psychological autopsy studies of suicide in Africa [102, 103], self-harm and suicide among women has been interpreted as protestation against socially sanctioned abuse and oppressive control, while men’s self-harm and suicide represent a quest for lost masculinity.

### Strengths and limitations of this review

Our comprehensive search strategy identified a substantial literature in a previously under-reviewed geographical area. Of the 74 studies, 12 (16·2%) were peer-reviewed articles exclusively indexed in the African regional academic databases searched. These 12 papers represent a valuable addition to previous reports covering parts of sub-Saharan Africa [10].

Our categorisation of the factors associated with self-harm into personal, family, school, interpersonal level factors was motivated by the wide variation and the general lack of meaningful classification of these factors across the studies and is not without limitations. For example, in terms of the factors associated with self-harm, there is likely to be significant inherent interdependence between, for example, personal level factors and family level factors – family factors could be influencing the onset of the personal level factors and vice-versa – or between personal, school and interpersonal levels. The categorisation into intrapersonal and interpersonal factors too is therefore likely to be subject to considerable interdependence.

### Future directions

Future studies should explore the prevalence estimates of self-harm among young people in non-clinical contexts such as the community and schools [6–8]. Participants in such prevalence studies should include other minority and vulnerable groups of young people including the homeless, and other out-of-school children and youth; lesbian, gay, bisexual, and transgender (LGBT) youth; orphans, and other children and youth in especially difficult circumstances including those with disability and in juvenile detention, who are often unrepresented or under-represented in population based studies on issues affecting young people [27, 33]. Recently, evidence of school-based studies from sub-Saharan Africa – and across the African continent, generally – indicates that the population of young people reporting LGBT and other sexual minority orientation is growing [104]. However, studies on their (mental) health needs are limited [99, 100].

Recent systematic reviews and primary studies from high-income countries indicate that street-connected children and adolescents represent a good case example of a high-risk group whose self-harm has received inadequate attention in the recent research literature [105]. In carrying out future studies, researchers should clearly define self-harm and more importantly, present to participants the operational definition used in the study, in order to facilitate recall and accurate responses.

More examination is needed of risk-factors for self-harm, not least in attempting to identify the temporal sequence of reported associations to help clarify interdependence between such factors. Future research (including qualitative studies) should also consider exploring factors such as social support, parenting styles, and school climate, which serve to protect young people in sub-Saharan Africa from engaging in self-harm. Such research can inform programmes aimed at strengthening protective and promotive factors within families and schools, and at local community levels can have significant positive effects on improving the developmental outcomes of vulnerable young people [106].

Too much research into risk and protective factors in self-harm has used concepts and measures developed in high income countries. We need to know more about specific features of life in sub-Saharan countries.

## Conclusion

Together, the studies in this review suggest that self-harm is a public (mental) health challenge in young people across countries within sub-Saharan Africa. Given what we know about the link between self-harm and poor mental health, impaired social function and increased suicide risk, more research into the epidemiology, causes and treatment of self-harm in this setting is justified. Too few studies from too few countries have examined the methods of self-harm, risks, protective factors, and the reasons associated with self-harm from a culturally and socially sensitive perspective. The findings of the reviewed studies were overly influenced by the use of pre-existing Western derived models and measures.

## Supplementary information

**Additional file 1.** PRISMA flow chart.

**Additional file 2.** PRISMA checklist.

**Additional file 3.** Search strategies.

**Additional file 4.** Authors contacted.

**Additional file 5.** Sources of included studies.

**Additional file 6.** Methods and designs used by studies.

**Additional file 7.** Methodological quality ratings of studies.

## Data Availability

An unpublished protocol guiding this review was completed in June 2016 by following PRISMA-P statement [107]. A copy of the protocol is available from the first author on reasonable request. All data generated or analysed during this study are included in this published article and its supplementary information files.

## Abbreviations

IQR: interquartile range
MMAT: mixed method appraisal tool
PRISMA: Preferred Reporting Items for Systematic Reviews and Meta-Analyses
PRISMA-P: Preferred Reporting Items for Systematic Reviews and Meta-Analysis protocols
SA-ETD: South African national Electronic Theses and Dissertations
WHO: World Health Organization
UNICEF: United Nations International Children’s Emergency Fund
UNDP: United Nations Development Programme
