# Supplementary material for: Self-harm with suicidal and non-suicidal intent in young people in sub-Saharan Africa: a systematic review
Source: BMC Psychiatry. 2020 May 14;20:234. doi: 10.1186/s12888-020-02587-z (PMC7222461; doi:10.1186/s12888-020-02587-z)
Supplement: Supplementary file 1 — Additional file 1. PRISMA flow chart. [file 12888_2020_2587_MOESM1_ESM.docx]

**Additional file 1.** PRISMA flow chart

Papers identified through database searching

(**n = 9,371**):

MEDLINE (n = 483)

PsycINFO (n = 83)

PubMED (n = 88)

African Journals OnLine (n= 600)

African Index Medicus (n = 8117)

Additional papers identified through other sources

**(n = 285):**

SA-ETD portal (n = 51)

Google scholar & Google search (n = 137)

Hand searching (n = 19)

Correspondence with authors (n = 63)

National & international reports (n = 15)

Potentially relevant papers **(n = 9,656)**

Duplicates removed (n = **1,238**)

Titles and abstracts of papers screened **(n = 8,418)**

Papers excluded after titles and abstract screening **(n = 7,864)**

Reasons for exclusion:

- Reviews on suicide and suicidal ideation (n=27)
- Suicide & medico-legal autopsy (n=58)
- Studies not related to self-harm (n=7,779)

**Identification**

**Screening**

Full text of papers assessed for eligibility **(n = 554)**

Full text of papers excluded **(n = 485)**

Reasons for exclusion:

- 90% of sample not within the 10-25 age band (n=31)
- Full text unavailable (n=7)
- No new data/duplicate of data (n=11)
- Self-harm defined, measured and reported as a composite variable including thoughts or plans, and attempts (n=12)
- Attitude studies of sample without self-harm history (n=8)
- Suicidal ideation (n=53)
- No suitable data related to adolescent self-harm (n=25)
- Adult sample (n=335)
- Adolescents living with HIV (n=3)

Additional titles through reference harvesting **(n = 17)**

**Eligibility**

Additional articles through reference harvesting excluded (**n =** **12**)

Additional papers through reference harvesting included **(n = 5)**

Papers included in review **(n = 74)**

**Included**
