# Supplementary material for: Self-harm with suicidal and non-suicidal intent in young people in sub-Saharan Africa: a systematic review
Source: BMC Psychiatry. 2020 May 14;20:234. doi: 10.1186/s12888-020-02587-z (PMC7222461; doi:10.1186/s12888-020-02587-z)
Supplement: Supplementary file 3 — Additional file 3. Search strategies. [file 12888_2020_2587_MOESM3_ESM.docx]

**Additional file 3. Search strategies**

**Ovid MELINE(R)**

| # | Query | Hits |
| --- | --- | --- |
| 1 | Self-harm OR self harm OR Self-harming behav* | 3863 |
| 2 | Suicide, Attempted/ OR Self-injurious behave*/ OR Suicide/ OR Self Mutilation/ OR self-mutilation | 53995 |
| 3 | Suicide ideation/ | 5253 |
| 4 | (Deliberate self-harm OR Delicate self-cutting OR Hair pulling OR Head banging OR Nonsuicidal self-injury OR Overdose OR Parasuicide OR Partial suicide OR Self-asphyxiation OR Self-burning OR Self-cutting OR Self-destruction OR Self-destructive behaviour OR Self-hitting OR Self-killing OR Self-laceration OR Self-poisoning OR Trichotillomania OR Wrist-cutting syndrome OR Wrist-slashing OR Non-fatal suicidal behav* OR Suicidal self-directed violence OR Non-suicidal self-directed violence OR Intentional self-harm).tw | 18555 |
| 5 | Drug overdose OR medication overdose OR Overdose.mp | 18096 |
| 6 | Antisuicid*.tw | 81 |
| 7 | Self-injur*.tw | 3444 |
| 8 | Suicid*.tw | 61465 |
| 9 | 1 OR 2 OR 3 OR 4 OR 5 OR 6 OR 7 OR 8 | 98652 |
| 10 | Adolescent/ | 1904124 |
| 11 | (Street adj3 child*).tw | 563 |
| 12 | (Street adj3 adolescen*).tw | 55 |
| 13 | (Street adj3 youth).tw | 311 |
| 14 | (rural adj3 child*).tw | 5012 |
| 15 | (urban child* OR urban adolescen* OR urban teens OR urban youth).tw | 2490 |
| 16 | school child*.tw | 19483 |
| 17 | (High school students OR secondary school students).tw | 8925 |
| 18 | (Homeless adolescen* OR homeless child* OR homeless youth).tw | 600 |
| 19 | (teenager* OR teen OR teens OR youth).tw | 63883 |
| 20 | (Orphans OR institutionalised children OR institutionalised children OR foster children OR children in residential care).tw | 1670 |
| 21 | Young adult/ | 709120 |
| 22 | (out-of-school child* OR out-of-school youth OR child* in especially difficult circumstance).tw | 121 |
| 23 | (street-connected young people OR street-connected children and young people OR street-connected children and youth OR street-connected children and adolescents OR child* with street connections OR child* in street situations OR child* working on the street OR child* living on the street).tw | 7 |
| 24 | 10 OR 11 OR 12 OR 13 OR 14 OR 15 OR 16 OR 17 OR 18 OR 19 OR 20 OR 21 OR 22 OR 23 | 2257506 |
| 25 | 9 AND 24 | 29502 |
| 26 | Africa South of the Sahara.mp. or exp "Africa South of the Sahara"/ | 189538 |
| 27 | (Angola OR Benin OR Dahomey OR Botswana OR Bechuanaland OR Burkina Faso OR Upper Volta OR Burundi OR Urundi OR Cameroon OR Cape Verde OR Cabo Verde OR Central African Republic OR Chad OR Comoros OR Congo OR Cote d'Ivoire OR Ivory Coast OR Democratic Republic of the Congo OR Zaire OR Equatorial Guinea OR Eritrea OR Ethiopia OR Abyssinia OR Gabon OR Gambia OR Ghana OR “Gold Coast” OR Guinea OR Guinea-Bissau OR Kenya OR Lesotho OR Basutoland OR Liberia OR Madagascar OR Malawi OR Mali OR Sudanese republic OR Mauritius OR Mozambique OR Namibia OR South-West Africa OR Niger OR Nigeria OR Rwanda OR Ruanda OR Sao Tome and Principe OR Senegal OR Seychelles OR Sierra Leone OR Somalia OR South Africa OR South Sudan OR eSwatini OR Swaziland OR United Republic of Tanzania OR Tanganyika OR Zanzibar OR Togo OR Uganda OR Zambia OR Northern Rhodesia OR Zimbabwe OR Southern Rhodesia OR German East Africa OR Western Sahara OR Central Africa OR Africa South of the Sahara OR West Africa OR Western Africa OR East Africa OR Eastern Africa OR Southern Africa OR sub-Saharan Africa OR subSaharan Africa).tw | 76166 |
| 28 | 26 OR 27 | 206248 |
| 29 | 25 AND 28 | 500 |
| 30 | limit 29 to yr=1950-2019 | 500 |

**Additional file 3.** (continued)

**PsycINFO**

| # | Query | Hits |
| --- | --- | --- |
| 1 | Self-harm OR self harm OR Self-harming behav* | 5405 |
| 2 | Suicide, Attempted/ OR Self-injurious behave*/ OR Suicide/ OR Self Mutilation/ OR self-mutilation | 33215 |
| 3 | Suicide ideation/ | 9338 |
| 4 | (Deliberate self-harm OR Delicate self-cutting OR Hair pulling OR Head banging OR Nonsuicidal self-injury OR Overdose OR Parasuicide OR Partial suicide OR Self-asphyxiation OR Self-burning OR Self-cutting OR Self-destruction OR Self-destructive behaviour OR Self-hitting OR Self-killing OR Self-laceration OR Self-poisoning OR Trichotillomania OR Wrist-cutting syndrome OR Wrist-slashing OR Non-fatal suicidal behav* OR Suicidal self-directed violence OR Non-suicidal self-directed violence OR Intentional self-harm).tw | 8376 |
| 5 | Drug overdose OR medication overdose OR Overdose.mp | 3424 |
| 6 | Antisuicid*.tw | 113 |
| 7 | Self-injur*.tw | 5943 |
| 8 | Suicid*.tw | 60410 |
| 9 | 1 OR 2 OR 3 OR 4 OR 5 OR 6 OR 7 OR 8 | 72003 |
| 10 | Adolescent.mp | 154000 |
| 11 | (Street adj3 child*).tw | 603 |
| 12 | (Street adj3 adolescen*).tw | 127 |
| 13 | (Street adj3 youth).tw | 502 |
| 14 | (rural adj3 child*).tw | 2008 |
| 15 | (urban child* OR urban adolescen* OR urban teens OR urban youth).tw | 2733 |
| 16 | school child*.tw | 17067 |
| 17 | (High school students OR secondary school students).tw | 34694 |
| 18 | (Homeless adolescen* OR homeless child* OR homeless youth).tw | 1245 |
| 19 | (teenager* OR teen OR teens OR youth).tw | 97564 |
| 20 | (Orphans OR institutionalised children OR institutionalised children OR foster children OR children in residential care).tw | 2524 |
| 21 | Young adult.mp | 13447 |
| 22 | (out-of-school child* OR out-of-school youth OR child* in especially difficult circumstance).tw | 98 |
| 23 | (street-connected young people OR street-connected children and young people OR street-connected children and youth OR street-connected children and adolescents OR child* with street connections OR child* in street situations OR child* working on the street OR child* living on the street).tw | 10 |
| 24 | 10 OR 11 OR 12 OR 13 OR 14 OR 15 OR 16 OR 17 OR 18 OR 19 OR 20 OR 21 OR 22 OR 23 | 274056 |
| 25 | 9 AND 24 | 11594 |
| 26 | Africa South of the Sahara.mp | 25 |
| 27 | (Angola OR Benin OR Dahomey OR Botswana OR Bechuanaland OR Burkina Faso OR Upper Volta OR Burundi OR Urundi OR Cameroon OR Cape Verde OR Cabo Verde OR Central African Republic OR Chad OR Comoros OR Congo OR Cote d'Ivoire OR Ivory Coast OR Democratic Republic of the Congo OR Zaire OR Equatorial Guinea OR Eritrea OR Ethiopia OR Abyssinia OR Gabon OR Gambia OR Ghana OR “Gold Coast” OR Guinea OR Guinea-Bissau OR Kenya OR Lesotho OR Basutoland OR Liberia OR Madagascar OR Malawi OR Mali OR Sudanese republic OR Mauritius OR Mozambique OR Namibia OR South-West Africa OR Niger OR Nigeria OR Rwanda OR Ruanda OR Sao Tome and Principe OR Senegal OR Seychelles OR Sierra Leone OR Somalia OR South Africa OR South Sudan OR eSwatini OR Swaziland OR United Republic of Tanzania OR Tanganyika OR Zanzibar OR Togo OR Uganda OR Zambia OR Northern Rhodesia OR Zimbabwe OR Southern Rhodesia OR German East Africa OR Western Sahara OR Central Africa OR Africa South of the Sahara OR West Africa OR Western Africa OR East Africa OR Eastern Africa OR Southern Africa OR sub-Saharan Africa OR subSaharan Africa).tw | 20787 |
| 28 | 26 OR 27 | 20787 |
| 29 | 25 AND 28 | 83 |
| 30 | limit 29 to yr=1950-2019 | 83 |

**Additional file 3.** (continued)

**PubMed**

| Search | Query | Hits |
| --- | --- | --- |
| # 1 | Antisuicid* OR Attempted suicide OR Deliberate self-harm OR Delicate self-cutting OR Hair pulling OR Head banging OR Nonsuicidal self-injury OR Overdose OR Parasuicide OR Partial suicide OR Self-asphyxiation OR Self-burning OR Self-cutting OR Self-destruct* OR Self-harm* OR Self-hitting OR Self-injur* OR Self-killing OR Self-laceration OR Self-mutilation OR Self-poisoning OR Suicid* OR Trichotillomania OR wrist-cutting OR Wrist-cutting syndrome OR Wrist-slashing OR self harm OR Self-harming behav* OR “self mutilation” OR Non-fatal suicidal behav* OR “Suicidal self-directed violence” OR “Non-suicidal self-directed violence” OR “Intentional self-harm” OR Self-injurious behav* | 120756 |
| # 2 | Adolescen* OR Child* OR Students OR Teen* OR “Young adults” OR youth OR Orphans OR institutionalised children OR institutionalised children OR foster children OR children in residential care OR out-of-school child* OR out-of-school youth OR child* in especially difficult circumstance OR street-connected young people OR street-connected children and young people OR street-connected children and youth OR street-connected children and adolescents OR child* with street connections OR child* in street situations OR child* working on the street OR child* living on the street OR “High school students” OR “secondary school students” | 12453 |
| # 3 | Angola OR Benin OR Dahomey OR Botswana OR Bechuanaland OR Burkina Faso OR Upper Volta OR Burundi OR Urundi OR Cameroon OR Cape Verde OR Cabo Verde OR Central African Republic OR Chad OR Comoros OR Congo OR Cote d'Ivoire OR Ivory Coast OR Democratic Republic of the Congo OR Zaire OR Equatorial Guinea OR Eritrea OR Ethiopia OR Abyssinia OR Gabon OR Gambia OR Ghana OR “Gold Coast” OR Guinea OR Guinea-Bissau OR Kenya OR Lesotho OR Basutoland OR Liberia OR Madagascar OR Malawi OR Mali OR Sudanese republic OR Mauritius OR Mozambique OR Namibia OR South-West Africa OR Niger OR Nigeria OR Rwanda OR Ruanda OR Sao Tome and Principe OR Senegal OR Seychelles OR Sierra Leone OR Somalia OR South Africa OR South Sudan OR eSwatini OR Swaziland OR United Republic of Tanzania OR Tanganyika OR Zanzibar OR Togo OR Uganda OR Zambia OR Northern Rhodesia OR Zimbabwe OR Southern Rhodesia OR German East Africa OR Western Sahara OR Central Africa OR Africa South of the Sahara OR West Africa OR Western Africa OR East Africa OR Eastern Africa OR Southern Africa OR sub-Saharan Africa OR subSaharan Africa | 518657 |
| # 4 | # 1 AND # 2 AND # 3 | 35 |
| # 5 | Filters activated: Publication date from 1950/01/01 to 2019/08/31, Humans, Female, Male, Child: 6-12 years, Adolescent: 13-18 years, Adult: 19+ years, Young Adult: 19-24 years, Adult: 19-44 years | 29 |

**African Journals OnLine (AJOL)**

| Search | Query | Hits |
| --- | --- | --- |
| # 1 | self-harm OR self harm OR deliberate self-harm | 23 |
| # 2 | self-injury OR nonsuicidial self-injury OR self-cutting OR self-mutilation OR self-directed violence | 500 |
| # 3 | suicide OR attempted suicide OR suicide attempt OR non-fatal suicidal behaviour | 16 |
| # 4 | parasuicide OR self-poisoning OR overdose | 57 |
| # 5 | self-burning OR self-immolation OR self-laceration | 4 |
|  | Total: | 600 |

**African Index Medicus (AIM) search strategy**

| Search | Query | Hits |
| --- | --- | --- |
| # 1 | self-harm OR self harm OR deliberate self-harm OR self-injury OR nonsuicidial self-injury OR self-cutting OR self-mutilation OR self-directed violence OR suicide OR attempted suicide OR suicide attempt OR non-fatal suicidal behaviour OR parasuicide OR self-poisoning OR overdose OR self-burning OR self-immolation OR self-laceration | 8117 |
