# Supplementary material for: Self-harm with suicidal and non-suicidal intent in young people in sub-Saharan Africa: a systematic review
Source: BMC Psychiatry. 2020 May 14;20:234. doi: 10.1186/s12888-020-02587-z (PMC7222461; doi:10.1186/s12888-020-02587-z)
Supplement: Supplementary file 4 — Additional file 4. Authors contacted. [file 12888_2020_2587_MOESM4_ESM.docx]

**Additional file 4. Authors contacted**

| **Author / Researcher** | **Contact** |
| --- | --- |
| Prof. Heidi Marie Hjelmeland | **Institution:** Department of Mental Health, Faculty of Medicine and Health Sciences, Norwegian University of Science and Technology  **Email:** heidi.hjelmeland@ntnu.no  **URL:** https://www.ntnu.edu/employees/heidi.hjelmeland |
| Prof. Eugene Kinyanda | **Institution:** MRC/UVRI Uganda Research Unit on AIDS  **Email:** Eugene.Kinyanda@mrcuganda.org  **URL:** http://www.mrcuganda.org/staff/professor-eugene-kinyanda |
| Prof. Birthe Loa Knizek | **Institution:** Department of Mental Health, Faculty of Medicine and Health Sciences, Norwegian University of Science and Technology  **Email:** birthe.l.knizek@ntnu.no  **URL:** http://www.ntnu.edu/employees/birthe.l.knizek |
| Prof. Stephanie Burrows | **Institution:** **Technical Officer,** WHO Department for the Management of Noncommunicable Diseases, Disability, Violence and Injury Prevention (NVI)  **Email:** burrowss@who.int  **URL:** http://www.who.int/ncds/management/burrows/en/ |
| Prof. Charity Sylvia Akotia | **Institution:** Department of Psychology, School of Social Sciences University of Ghana, Legon.  **Email:** sakotia@ug.edu.gh  **URL:** http://www.ug.edu.gh/psychology/staff/charity-sylvia-akotia-0 |
| Prof Derege Kebede | **Institution:** Johns Hopkins Bloomberg School of Public Health  **Email:** dkebede1@jhu.edu  **URL:** https://www.jhsph.edu/faculty/directory/profile/3362/derege-kebede |
| Dr. Atalay Alem | **Institution:** Department of Psychiatry, University of Toronto  **Email:** atalay.alem@gmail.com  **URL:** http://www.psychiatry.utoronto.ca/people/dr-atalay-alem/ |
| Prof Anthony Lingum Pillay | **Institution:** Department of Behavioural Medicine, University of Kwazulu-Natal, South Africa  **Email:** anthony.pillay@kznhealth.gov.za  **URL:** http://behavmed.ukzn.ac.za/Staff/ProfessorAnthonyLPillay.aspx |
| Prof Douglas R. Wassenaar | **Institution:** School of Applied Human Sciences, University of Kwazulu-Natal, South Africa  **Email:** wassenaar@ukzn.ac.za  **URL:** http://sahs.ukzn.ac.za/staff-profile/psychology/douglas-r-wassenaar.aspx |
| Prof Olayinka Olusola Omigbodun | **Institution:** College of Medicine, University of Ibadan, Nigeria  **Email:** olayinka.omigbodun@gmail.com  **URL:** http://com.ui.edu.ng/index.php/prof-olayinka-o-omigbodun |
| Prof Lauren G. Wild | **Institution:** Department of Psychology, University of Cape Town, South Africa  **Email:** Lauren.Wild@uct.ac.za  **URL:** http://www.psychology.uct.ac.za/psy/staff/seniorlecturers/laurenwild |
| Prof Monica Haavisto Swahn | **Institution:** School of Public Health, Georgia State University, Atlanta  **Email:** mswahn@gsu.edu  **URL:** http://publichealth.gsu.edu/profile/monica-swahn/ |
| Dr. Kwaku Oppong Asante | **Institution:** Department of Psychology, University of Ghana, Accra.  **Email:** koppongasante@ug.edu.gh  **URL:** https://www.researchgate.net/profile/Kwaku_Oppong_Asante |
| Prof Joseph Osafo | **Institution:** Department of Psychology, University of Ghana, Accra.  **Email:** josafo@ug.edu.gh  **URL:** http://www.ug.edu.gh/psychology/staff/joseph-osafo |
| Prof Lewis Aptekar | Institution: College of Education, San Jose State University, San Jose, California.  Email: lewis.aptekar@sjsu.edu  URL: http://www.sjsu.edu/faculty/laptekar/ |
| Prof. Tatek Abebe | **Institution:** Department of Education and lifelong learning, Norwegian University of Science and Technology, Trondheim, Norway  **Email:** tatek.abebe@ntnu.no  **URL:** https://www.ntnu.edu/employees/abebe |
| Dr. Tigest Ketsela Mengestu | **Institution:** WHO Representative – Country office, Swaziland.  **Email:** ketselat@who.int  **URL:** http://sz.one.un.org/content/unct/swaziland/en/home/agencies/who.html |
| Prof Alan J. Flisher Centre for Public Mental Health | **Institution:** Prof Alan J. Flisher Centre for Public Mental Health, South Africa  **Email:** gillian.hanslo@uct.ac.za / skeen@sun.ac.za  **URL:** http://www.cpmh.org.za/ |
| Dr. Jason Bantjes | **Institution:** Department of Psychology, Stellenbosch University, South Africa  **Email:** jbantjes@sun.ac.za  **URL:** https://www.sun.ac.za/english/faculty/arts/psychology/staff/academic-staff |
| Dr. Hilda N. Shilubane | **Institution:** Department of Advanced Nursing Science, University of Venda, South Africa.  **Email:** hilda.shilubane@univen.ac.za  **URL:** http://www.univen.ac.za/school-of-health-sciences/advanced-nursing-science/ |
