# Supplementary material for: Self-harm with suicidal and non-suicidal intent in young people in sub-Saharan Africa: a systematic review
Source: BMC Psychiatry. 2020 May 14;20:234. doi: 10.1186/s12888-020-02587-z (PMC7222461; doi:10.1186/s12888-020-02587-z)
Supplement: Supplementary file 5 — Additional file 5. Sources of included studies. [file 12888_2020_2587_MOESM5_ESM.docx]

**Additional file 5. Sources of included studies (n=74)**

| **Record** | **Category** | **n (%)** | **Author(s)** |
| --- | --- | --- | --- |
| Global school-based student health survey | National report | 1 (1.4) | Nyandindi (2017).^1^ |
| Postgraduate theses | Postgraduate theses | 7 (9.4) | Campbell (2012),^2^ Kritzinger (2018),^3^ Lippi (2014),^4^ Nanewortor (2011),^5^ Pretorius (2011),^6^ Sommer (2005),^7^ van Rooyen (2013).^8^ |
| Peer-reviewed published papers | Book chapter | 1 (1.4) | Sefa-Dedeh & Canetto (1992).^9^ |
|  | Indexed academic journal articles | 65 (87.8) | Akanni et al. (2017),^10^ Amare et al. (2018),^11^ Asante et al. (2017),^12^ Asante & Meyer-Weitz (2017),^13^ Baiden et al. (2019),^14^ Beekrum et al. (2011),^15^ Brittain et al. (2019),^16^ Carvalho et al. (2019),^17^ Cheng et al. (2014),^18^ Chinawa et al. (2014),^19^ Cluver et al. (2015),^20^ Cummins & Allwood (1984),^21^ Darré et al. (2019),^22^ Fine et al. (2012),^23^ Flisher, Ward et al. (2006),^24^ Flisher, Ziervogel et al. (1993),^25^ Gage (2013),^26^ Giru (2016),^27^ James et al. (2017),^28^ Kebede & Ketsela, (1993),^29^ Khuzwayo et al. (2018),^30^ Kinyanda et al. (2011),^31^ Konayagi, Oh et al. (2019),^32^ Konayagi, Stubbs et al. (2019),^33^ Liu et al. (2018),^34^ Madu & Matla (2003, 2004),^35,36^ Mashego & Madu (2009),^37^ Meissner & Bantjes (2017),^38^ Mhlongo & Peltzer (1999),^39^ Muula et al. (2013),^40^ Ng et al. (2015),^41^ Nguyen et al. (2019),^42^ OKoko et al. (2011),^43^ Omigbodun et al. (2008),^44^ Peltzer (2008),^45^ Peltzer et al. (2000),^46^ Peltzer & Pengpid (2017),^47^ Penning & Collings (2014),^48^ Pillay (1987, 1988),^49,50^ Pillay & Wassenaar (1991, 1997),^51,52^ Quarshie et al. (2019),^53^ Randall et al. (2014),^54^ Schlebusch (1985),^55^ Shaikh et al. (2016),^56^ Shayo & Lawala (2019),^57^ Shiferaw et al. (2006),^58^ Shilubane et al. (2012, 2013, 2014),^59-61^ Stansfeld et al. (2017),^62^ Swahn et al. (2012), ^63^ Thornton et al. (2019),^64^ Tolulope et al. (2019),^65^ Uddin et al. (2019),^66^ van der Wal & George (2018),^67^ van der Walt (2016),^68^ van Niekerk et al. (2012),^69^ Vancampfort et al. (2019),^70^ Vawda (2012),^71^ Wassenaar et al. (1998),^72^ Wild et al. (2004),^73^ Yéo-Tenena et al. (2010).^74^ |
| – | Total | 74 (100) | – |
