# Supplementary material for: Self-harm with suicidal and non-suicidal intent in young people in sub-Saharan Africa: a systematic review
Source: BMC Psychiatry. 2020 May 14;20:234. doi: 10.1186/s12888-020-02587-z (PMC7222461; doi:10.1186/s12888-020-02587-z)
Supplement: Supplementary file 6 — Additional file 6. Methods and designs used by studies. [file 12888_2020_2587_MOESM6_ESM.docx]

**Additional file 6. Methods and designs used by studies (n=74)**

| Broad Method. n (%) | Design. n (%) | Brief Description | Author(s) |
| --- | --- | --- | --- |
| Quantitative: 66 (89.2) | Case control: 1 (1.4) | Clinical case control study involving suicidal patients and a non-suicidal medical control and a non-suicidal community control. | Pillay & Wassenaar (1997).^52^ |
|  | Case series: 7 (9.4) | Quantitative descriptive content analysis of patient clinical records. | Cummins & Allwood (1984),^21^ Schlebusch (1985),^55^  Pillay (1987, 1988),^49,50^ Mhlongo & Peltzer (1999),^39^ Okoko et al. (2011),^43^ Yéo-Tenena et al. (2010).^74^ |
|  | Case study: 1 (1.4) | Quantitative descriptive analysis of structured interviews in hospital with out-patients who had history of attempted suicide. | Fine et al. (2012).^23^ |
|  | Cohort design: 1 (1.4) | Longitudinal repeated structured interviews (1 year apart) in selected households within community. | Cluver et al. (2015).^20^ |
|  | Cross-sectional: 56 (75.6) | Structured questionnaire survey given out in community / household | Asante & Meyer-Weitz (2017),^13^ Gage (2013),^26^ Kinyanda et al. (2011),^31^ Cheng et al. (2014),^18^ Ng et al. (2015),^41^ Nguyen et al. (2019),^42^ Thornton et al. (2019).^64^ |
|  |  | Structured questionnaire survey administered at a youth centre or charity facility | Brittain et al. (2019),^16^ Swahn et al. (2012).^63^ |
|  |  | Structured questionnaire survey given out at a hospital | Pillay & Wassenaar (1991).^51^ |
|  |  | Structured questionnaire survey given out at schools and universities/colleges | School (n = 40): Akanni et al. (2017),^10^ Amare et al. (2018),^11^ Asante et al. (2017),^12^ Baiden et al. (2019),^14^ Carvalho et al. (2019),^17^ Chinawa et al. (2014),^19^ Campbell, (2012),^2^ Darré et al. (2019),^22^ Flisher, Ward et al. (2006),^24^ Flisher, Ziervogel et al. (1993),^25^ Giru (2016),^27^ James et al. (2017),^28^ Kebede & Ketsela, (1993),^29^ Khuzwayo et al. (2018),^30^ Konayagi, Oh et al. (2019),^32^ Konayagi, Stubbs et al. (2019),^33^ Liu et al. (2018),^34^ Madu & Matla (2003, 2004),^35,36^ Mashego & Madu (2009),^37^ Muula et al. (2013),^40^ Nanewortor (2011),^5^ Nyandindi (2017),^1^ Omigbodun et al. (2008),^44^ Peltzer (2008),^45^ Peltzer et al. (2000),^46^ Peltzer & Pengpid (2017),^47^ Penning & Collings (2014),^48^ Randall et al. (2014),^54^ Shaikh et al. (2016),^56^ Shayo & Lawala (2019),^57^ Shilubane et al. (2013, 2014),^60,61^ Sommer (2005),^7^ Stansfeld et al. (2017),^62^ Tolulope et al. (2019),^65^ Uddin et al. (2019),^66^ van der Wal & George (2018),^67^ Vancampfort et al. (2019),^70^ Vawda (2012),^71^ Wild et al. (2004).^73^ University/College (n = 5): Lippi (2014),^4^ Quarshie et al. (2019),^53^ van der Walt (2016),^68^ van Niekerk et al. (2012),^69^ van Rooyen (2013).^8^ |
| Qualitative: 6 (8.1) | Case study design: 6 (8.1) | Qualitative in-depth interviews with attempted suicide survivors in a hospital and community. | Hospital (n = 4): Beekrum et al. (2011),^15^ Kritzinger (2018),^3^ Sefa-Dedeh & Canetto (1992),^9^ Wassenaar et al. (1998).^72^  Community (n = 1): Shilubane et al. (2012).^59^  University (n=1): Meissner & Bantjes (2017).^38^ |
| Mixed method: 2 (2.7) | Sequential design: 2 (2.7) | Structured quantitative questionnaire survey followed by qualitative focus-group discussion or individual interviews with selected participants. | Shiferaw et al. (2006),^58^ Pretorius (2011).^6^ |
