# Supplementary material for: Self-harm with suicidal and non-suicidal intent in young people in sub-Saharan Africa: a systematic review
Source: BMC Psychiatry. 2020 May 14;20:234. doi: 10.1186/s12888-020-02587-z (PMC7222461; doi:10.1186/s12888-020-02587-z)
Supplement: Supplementary file 7 — Additional file 7. Methodological quality ratings of studies. [file 12888_2020_2587_MOESM7_ESM.docx]

**Additional file 7.** **Methodological quality ratings of studies using the Mixed Method Appraisal Tool, MMAT** (Hong et al., 2018)^75,76^

| Screening questions (for all study types and designs included in review) | S1. | Are there clear research questions? |
| --- | --- | --- |
|  | S2. | Do the collected data allow to address the research questions? |

**Quantitative descriptive studies (retrospective chart reviews, case control studies, cohort designs, and cross-sectional survey designs)**

MMAT criteria for quantitative descriptive studies:

C1: Is the sampling strategy relevant to address the quantitative research question?

C2: Is the sample representative of the target population?

C3: Are the measurements appropriate?

C4: Is the risk of nonresponse bias low?

C5: Is the statistical analysis appropriate to answer the research question?

| Study Reference | Methodological Quality Criteria | | | | | Total  Score | Comments |
| --- | --- | --- | --- | --- | --- | --- | --- |
|  | C1 | C2 | C3 | C4 | C5 |  |  |
| Retrospective chart review (quantitative descriptive analysis of patient clinical records): | | | | | |  |  |
| Cummins & Allwood (1984)^21^ | Yes | Can’t tell | Can’t tell | Yes | Yes | 3 | Size of target population not reported.  Suicide attempt was established by an unspecified diagnostic case ascertainment at hospital admission. |
| Schlebusch (1985)^55^ | Yes | Yes | Can’t tell | Yes | Yes | 4 | Self-destructive behaviour was established by an unspecified diagnostic case ascertainment at hospital admission. |
| Okoko et al. (2011)^43^ | Yes | Yes | Can’t tell | Yes | Yes | 4 | Suicidal behaviour was established by an unspecified diagnostic case ascertainment at hospital admission. |
| Pillay (1987)^49^ | Yes | Can’t tell | Can’t tell | Yes | Yes | 3 | Size of target population not reported.  Parasuicide was established by an unspecified diagnostic case ascertainment at hospital admission. |
| Pillay (1988)^50^ | Yes | Can’t tell | Can’t tell | Yes | Yes | 3 | Size of target population not reported.  Parasuicide was established by an unspecified diagnostic case ascertainment at hospital admission. |
| Mhlongo & Peltzer (1999)^39^ | Yes | Can’t tell | Can’t tell | Yes | Yes | 3 | Size of target population not reported.  Parasuicide was established by an unspecified diagnostic case ascertainment at hospital admission. |
| Yéo-Tenena et al. (2010)^74^ | Yes | Yes | Can’t tell | Yes | Yes | 4 | Suicidal behaviour was established by an unspecified diagnostic case ascertainment at hospital admission. |

**Additional file 7.** (continued)

| Study Reference | Methodological Quality Criteria | | | | | Total  Score | Comments |
| --- | --- | --- | --- | --- | --- | --- | --- |
|  | C1 | C2 | C3 | C4 | C5 |  |  |
| Cross-sectional survey (questionnaires given out at school, university, or community): | | | | | |  |  |
| Asante & Meyer-Weitz (2017)^13^ | Yes | Can’t tell | Yes | Yes | Yes | 4 | Size of the target population and sample size determination strategy were not reported. Authors pre-screened candidate factors using bivariate tests and rejected from the final multivariable logistic regression model factors which showed no statistically significant bivariate relationship with the outcome variable. |
| Cheng et al. (2014)^18^ | Yes | Can’t tell | Yes | Yes | Yes | 4 | Size of the target population and sample size determination strategy were not reported. |
| Gage (2013)^26^ | Can’t tell | Yes | Yes | Yes | Yes | 4 | Sampling strategy not described |
| Kinyanda et al. (2011)^31^ | Yes | Yes | Yes | Yes | Yes | 5 |  |
| Ng et al. (2015)^41^ | Yes | Yes | Yes | Yes | Yes | 5 |  |
| Thornton et al. (2019)^64^ | Can’t tell | Can’t tell | Yes | Can’t tell | Yes | 2 | Size of the target population, sample size determination strategy, and sampling process were insufficiently described. Response rate not reported. |
| Swahn et al. (2012)^63^ | Yes | Yes | Yes | Yes | Yes | 4 | Size of the target population and sample size determination strategy were not reported.  Authors pre-screened candidate factors using bivariate tests and rejected from the final multivariable logistic regression model factors which showed no statistically significant bivariate relationship with the outcome variable. |
| Fine et al. (2012)^23^ | Yes | Can’t tell | Can’t tell | Yes | Yes | 3 | Size of target population and sample size determination strategy were not reported.  Suicide attempt was established by an unspecified diagnostic case ascertainment at hospital admission. |
| Pillay & Wassenaar (1991)^51^ | Yes | Can’t tell | Can’t tell | Yes | Yes | 3 | Size of target population and sample size determination strategy were not reported.  Suicide attempt was established by an unspecified diagnostic case ascertainment at hospital admission. |
| Akanni et al. (2017)^10^ | Yes | Can’t tell | Yes | Can’t tell | Can’t tell | 2 | Size of the target population was not reported.  Response rate was not reported.  Specific statistical analysis tools and process were not described. |
| Amare et al. (2018)^11^ | Yes | Yes | Yes | Yes | Yes | 4 | Authors pre-screened candidate factors using bivariate tests and rejected from the final multivariable logistic regression model factors which showed no statistically significant bivariate relationship with the outcome variable. |
| Asante et al. (2017)^12^ | Yes | Yes | Yes | Yes | Yes | 5 | Authors pre-screened candidate factors using bivariate tests and rejected from the final multivariable logistic regression model factors which showed no statistically significant bivariate relationship with the outcome variable. |
| Baiden et al. (2019)^14^ | Yes | Yes | Yes | Yes | Yes | 5 |  |
| Brittain et al. (2019)^16^ | Yes | Can’t tell | Yes | Yes | Yes | 4 |  |
| Carvalho et al. (2019)^17^ | Yes | Yes | Yes | Yes | Yes | 5 |  |
| Campbell (2012)^2^ | Yes | Can’t tell | Yes | Can’t tell | Yes | 3 | Size of the target population was not reported.  Sampling strategy was not described. |
| Chinawa et al. (2014)^19^ | Yes | Can’t tell | Yes | Can’t tell | Can’t tell | 2 | Size of the target population was not reported.  Response rate was not reported.  Specific statistical analysis tools and process were not described. |

**Additional file 7.** (continued)

| Study Reference | Methodological Quality Criteria | | | | | Total  Score | Comments |
| --- | --- | --- | --- | --- | --- | --- | --- |
|  | C1 | C2 | C3 | C4 | C5 |  |  |
| Cross-sectional survey (continued): | | | | | |  |  |
| Darré et al. (2019)^22^ | Yes | Can’t tell | Yes | Can’t tell | Yes | 3 | Size of the target population was not reported.  Response rate was not reported. |
| Flisher et al. (1993)^25^ | Yes | Can’t tell | Yes | Can’t tell | Can’t tell | 2 | Size of the target population was not reported.  Response rate was not reported.  Specific statistical analysis tools and process were not described. |
| Flisher et al. (2006)^24^ | Yes | Can’t tell | Yes | Can’t tell | Yes | 3 | Size of the target population was not reported.  Response rate was not reported. |
| Giru (2016)^27^ | Can’t tell | Can’t tell | Yes | Yes | Yes | 3 | sample size determination strategy, and sampling process were not described. Author pre-screened candidate factors using bivariate tests and rejected from the final multivariable logistic regression model factors which showed no statistically significant bivariate relationship with the outcome variable. |
| James et al. (2017)^28^ | Yes | Yes | Yes | Can’t tell | Yes | 4 | Response rate was not reported.  Authors pre-screened candidate factors using bivariate tests and rejected from the final multivariable logistic regression model factors which showed no statistically significant bivariate relationship with the outcome variable. |
| Kebede & Ketsela (1993)^29^ | Yes | Yes | Yes | Yes | Yes | 5 |  |
| Khuzwayo et al. (2018)^30^ | Yes | Can’t tell | Yes | Can’t tell | Yes | 3 | Size of the target population was not reported.  Response rate was not reported. |
| Koyanagi, Oh et al. (2019)^32^ | Yes | Yes | Yes | Yes | Yes | 5 |  |
| Koyanagi, Stubbs et al. (2019)^33^ | Yes | Yes | Yes | Yes | Yes | 5 |  |
| Lippi (2014)^4^ | Yes | Can’t tell | Yes | Can’t tell | Yes | 3 | Size of the target population, sample size determination strategy, and sampling process were insufficiently described. Response rate not reported. |
| Liu et al. (2018)^34^ | Yes | Yes | Yes | Yes | Yes | 5 |  |
| Madu & Matla (2003)^35^ | Yes | Can’t tell | Yes | Yes | Yes | 4 | Size of the target population was not reported. |
| Madu & Matla (2004)^36^ | Yes | Can’t tell | Yes | Yes | Yes | 4 | Size of the target population was not reported. |
| Mashego & Madu (2009)^37^ | Yes | Can’t tell | Yes | Yes | Yes | 4 | Size of the target population was not reported. |
| Muula et al. (2013)^40^ | Yes | Yes | Yes | Yes | Yes | 5 | Authors used an automated variable selection process (backward elimination) to select candidate factors included in the multivariable regression model. |
| Nanewortor (2011)^5^ | Yes | Yes | Yes | Can’t tell | Yes | 4 | Response rate was not reported. |
| Nguyen et al. (2019)^42^ | Yes | Yes | Yes | Yes | Yes | 5 |  |
| Nyandindi (2017)^1^ | Yes | Yes | Yes | Yes | Yes | 5 |  |
| Omigbodun et al. (2008)^44^ | Yes | Can’t tell | Yes | Yes | Yes | 4 | Size of the target population and sample size determination strategy were not reported.  Authors pre-screened candidate factors using bivariate tests and rejected from the final multivariable logistic regression model factors which showed no statistically significant bivariate relationship with the outcome variable. |
| Peltzer (2008)^45^ | Yes | Can’t tell | Yes | Yes | Yes | 4 | Size of the target population was not reported. |
| Peltzer et al. (2000)^46^ | Yes | Can’t tell | Yes | Can’t tell | Yes | 3 | Size of the target population and sampling procedure were not described.  Response rate was not reported. |

**Additional file 7.** (continued)

| Study Reference | Methodological Quality Criteria | | | | | Total  Score | Comments |
| --- | --- | --- | --- | --- | --- | --- | --- |
|  | C1 | C2 | C3 | C4 | C5 |  |  |
| Cross-sectional survey (continued): | | | | | |  |  |
| Peltzer & Pengpid (2017)^47^ | Yes | Can’t tell | Yes | Yes | Yes | 4 |  |
| Penning & Collings (2014)^48^ | Yes | Yes | Yes | Yes | Yes | 5 |  |
| Quarshie et al. (2019)^53^ | Yes | Yes | Yes | Yes | Yes | 5 |  |
| Randall et al. (2014)^54^ | Yes | Yes | Yes | Yes | Yes | 5 |  |
| Shaikh et al. (2016)^56^ | Yes | Yes | Yes | Yes | Yes | 5 | Authors pre-screened candidate factors using bivariate tests and rejected from the final multivariable logistic regression model factors which showed no statistically significant bivariate relationship with the outcome variable. |
| Shayo & Lawala. (2019)^57^ | Yes | Yes | Yes | Yes | Yes | 5 |  |
| Shilubane et al. (2013)^60^ | Yes | Yes | Yes | Yes | Yes | 5 |  |
| Shilubane et al. (2014)^61^ | Yes | Can’t tell | Yes | Yes | Yes | 4 | Size of the target population and sample size determination strategy were not reported.  Authors pre-screened candidate factors using bivariate tests and rejected from the final multivariable logistic regression model factors which showed no statistically significant bivariate relationship with the outcome variable. |
| Sommer (2005)^7^ | Can’t tell | Can’t tell | Yes | Can’t tell | Yes | 2 | Size of the target population, sample size determination strategy, and sampling process were insufficiently described.  Response rate not reported. |
| Stansfeld et al. (2017)^62^ | Yes | Can’t tell | Yes | Yes | Yes | 4 | Size of the target population was not reported. |
| Tolulope et al. (2019)^65^ | Yes | Yes | Yes | Yes | Can’t tell | 4 | The final logistic regression modelling and the bases for inclusion of potential correlates were insufficiently described. |
| Uddin et al. (2019)^66^ | Yes | Yes | Yes | Yes | Yes | 5 |  |
| van der Wal & George (2018)^67^ | Yes | Can’t tell | Yes | Can’t tell | Yes | 3 | Size of the target population, sample size determination strategy, and sampling process were insufficiently described.  Response rate not reported. |
| Vancampfort et al. (2019)^70^ | Yes | Yes | Yes | Yes | Yes | 5 |  |
| Vawda (2012)^71^ | Yes | Can’t tell | Yes | Can’t tell | Yes | 3 | Size of the target population, sample size determination strategy, and sampling process were insufficiently described.  Response rate not reported. |
| van der Walt (2016)^68^ | Yes | Can’t tell | Yes | Can’t tell | Yes | 3 | Size of the target population, sample size determination strategy, and sampling process were insufficiently described.  Response rate not reported. |
| van Niekerk et al. (2012)^69^ | Yes | Can’t tell | Yes | Can’t tell | Yes | 3 | Size of the target population, sample size determination strategy, and sampling process were insufficiently described.  Response rate not reported. |
| van Rooyen (2013)^8^ | Yes | Can’t tell | Yes | Can’t tell | Yes | 3 | Size of the target population, sample size determination strategy, and sampling process were insufficiently described.  Response rate not reported. |
| Wild et al. (2004)^73^ | Yes | Can’t tell | Yes | Yes | Yes | 4 | Size of the target population was not reported. |

**Additional file 7.** (continued)

| Study Reference | Methodological Quality Criteria | | | | | Total  Score | Comments |
| --- | --- | --- | --- | --- | --- | --- | --- |
|  | C1 | C2 | C3 | C4 | C5 |  |  |
| Case control study: |  |  |  |  |  |  |  |
| Pillay & Wassenaar (1997)^52^ | Yes | Yes | Yes | Yes | Yes | 5 |  |
| Cohort study: |  |  |  |  |  |  |  |
| Cluver et al. (2015)20 | Can’t tell | Yes | Yes | Yes | Yes | 4 | Size of target population not reported |

**Qualitative studies**

MMAT criteria for qualitative studies:

C1: Is the qualitative approach appropriate to answer the research question?

C2: Are the qualitative data collection methods adequate to address the research question?

C3: Are the findings adequately derived from the data?

C4: Is the interpretation of results sufficiently substantiated by data?

C5: Is there coherence between qualitative data sources, collection, analysis and interpretation?

| Study Reference | Methodological Quality Criteria | | | | | Total  Score | Comments |
| --- | --- | --- | --- | --- | --- | --- | --- |
|  | C1 | C2 | C3 | C4 | C5 |  |  |
| Sefa-Dedeh & Canetto (1992)^9^ | Yes | Yes | Yes | Yes | Can’t tell | 4 | Specific method of data analysis is neither reported nor described. |
| Wassenaar et al. (1998)^72^ | Yes | Yes | Yes | Yes | Can’t tell | 4 | Specific method of data analysis is neither reported nor described. |
| Beekrum et al. (2011)^15^ | Yes | Yes | Yes | Yes | Yes | 5 |  |
| Shilubane et al. (2012)^59^ | Yes | Yes | Yes | Yes | Can’t tell | 4 | Specific method of data analysis is not reported. |
| Meissner & Bantjes (2017)^38^ | Yes | Yes | Yes | Yes | Yes | 5 |  |
| Kritzinger (2018)^3^ | Yes | Yes | Yes | Yes | Yes | 5 |  |

**Mixed methods studies**

MMAT criteria for mixed methods studies:

C1: Is there an adequate rationale for using a mixed methods design to address the research question?

C2: Are the different components of the study effectively integrated to answer the research question?

C3: Are the outputs of the integration of qualitative and quantitative components adequately interpreted?

C4: Are divergences and inconsistencies between quantitative and qualitative results adequately addressed?

C5: Do the different components of the study adhere to the quality criteria of each tradition of the methods involved?

| Study Reference | Methodological Quality Criteria | | | | | Total  Score | Comments |
| --- | --- | --- | --- | --- | --- | --- | --- |
|  | C1 | C2 | C3 | C4 | C5 |  |  |
| Shiferaw et al. (2006)^58^ | Yes | No | Yes | No | No | 2 | No information on the sampling, procedure and the analysis strategy/ process for the qualitative bit. Insufficient information on the population studied. |
| Pretorius (2011)^6^ | Yes | Yes | No | No | Yes | 3 | Divergences and inconsistencies between quantitative and qualitative results were not addressed. |
